# ScRNA-seq unveils the functional characteristics of glioma-associated macrophages and the regulatory effects of chlorogenic acid on the immune microenvironment—a study based on mouse models and clinical practice

**DOI:** 10.3389/fimmu.2024.1494806

**Published:** 2025-01-10

**Authors:** Jiachen Wang, Shenglan Li, Yuxiao Chen, Jinyi Chen, Can Wang, Zhuang Kang, Mengqian Huang, Zehao Cai, Yuxiang Fan, Yanjie Lan, Yumeng Yu, Ruijing Bai, Feng Chen, Jiandong Jiang, Wenbin Li

**Affiliations:** ^1^ Department of Neuro-oncology, Cancer Center, Beijing Tiantan Hospital, Capital Medical University, Beijing, China; ^2^ Chinese Institute for Brain Research, Capital Medical University, Beijing, China; ^3^ Department of Hematology, Xuanwu Hospital, Capital Medical University, Beijing, China; ^4^ Department of Neurosurgery, Capital Medical University Xuanwu Hospital, Beijing, China; ^5^ Institute of Medicinal Biotechnology, Chinese Academy of Medical Sciences and Peking Union Medical College, Beijing, China

**Keywords:** chlorogenic acid (5-caffeoylquinic acid), glioma, tumor-associated macrophages (TAM), microglia, immune microenvironment (IME), single-cell RNA (scRNA) sequencing, network pharmacology (NP)

## Abstract

**Introduction:**

Glioma is the most common primary malignant brain tumor. Despite advances in surgical techniques and treatment regimens, the therapeutic effects of glioma remain unsatisfactory. Immunotherapy has brought new hope to glioma patients, but its therapeutic outcomes are limited by the immunosuppressive nature of the tumor microenvironment (TME). This study aimed to reveal the subpopulations and functional characteristics of tumor-associated macrophages (TAMs) and explore the regulatory effects of chlorogenic acid (CHA) on the immune microenvironment, as well as its potential for clinical application.

**Methods:**

In this study, CHA was used in model mice. ScRNA - seq analysis was conducted to elucidate the differentiation trajectories and functional characteristics of bone marrow - derived monomacrophages (BMDMs) and microglia. A PPI and molecular docking model were constructed using the target prediction database. A case of a patient treated with CHA was reviewed.

**Results:**

CHA slowed tumor growth in model mice and extended the survival time of mice. It enhanced the antigen - presenting function of macrophages and T - cell immune activation - related gene expression, activated microglia through the JAK - STAT pathway, and improved the antitumor functions. The good affinity of CHA with STAT1 was confirmed. The patient treated with CHA survived for 5 years and 6 months, achieved partial remission (PR) after 9 months of treatment, and remained alive without any new symptoms or toxic side effects. Our study revealed the subtypes and differentiation trajectories of TAMs. CHA significantly improved the immune microenvironment of glioma by modulating the function of BMDMs and microglia.

**Discussion:**

This study may provide new insights into targeting the regulation of TME and offer theoretical and practical support for the clinical application of CHA. The results demonstrated the potential of CHA in improving the immune microenvironment and antitumor effects, which could have implications for future glioma treatment strategies.

## Graphical Abstract

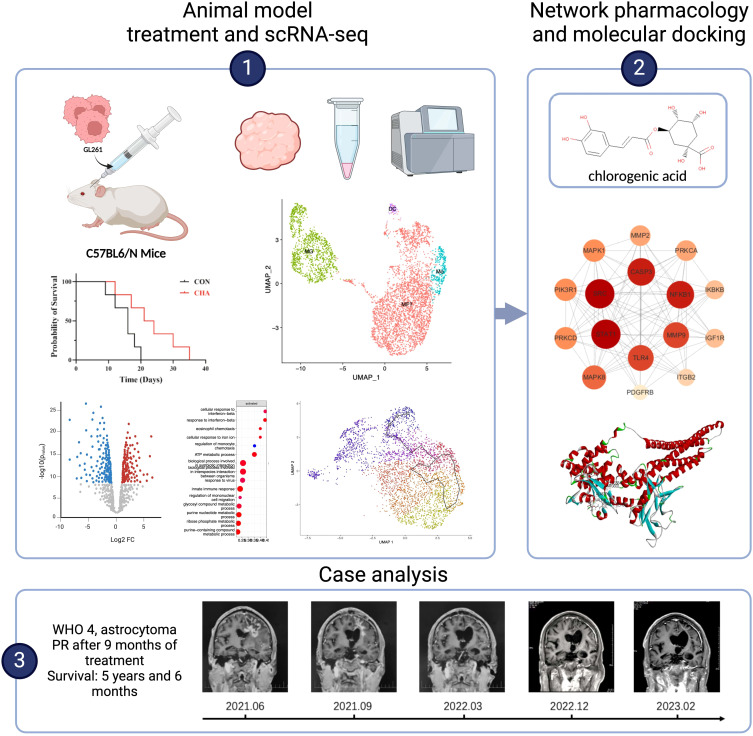

## 1 Introduction

Glioma is the most common primary malignant brain tumor among adults, accounting for approximately 30% of all primary brain tumors and 80% of malignant brain tumors (1). Glioblastoma (GBM) is the most aggressive subtype of glioma, for which the standard treatments primarily include surgical resection, radiotherapy, and chemotherapy. However, despite advancements in surgical techniques and treatment protocols, the prognosis for GBM remains poor (2). At present, temozolomide is the only specific drug for GBM treatment, but its efficacy is still not satisfactory.

In recent years, immunotherapy, as a novel therapeutic approach, has brought hope to GBM patients. The type of immunotherapy mainly includes immune checkpoint inhibitors (such as PD-1 and PD-L1 inhibitors/antibodies), chimeric antigen receptor T-cell therapy (CAR-T), and oncolytic virus therapy (3, 4). However, the efficacy of these treatment modalities is often limited by the highly immunosuppressive nature of the tumor microenvironment (TME) in GBM, coupled with the relatively low selective permeability of the blood-brain barrier and heterogeneity of tumor cells (3).

TME is composed of blood vessels surrounding tumor cells, immune cells, stromal cells, extracellular matrix, and other signaling molecules (5). It is recognized as a dynamic interplay of components that collectively respond to the growth needs of tumor cells and has become one of the main reasons for tumor progression and drug resistance (5). Among them, tumor-associated macrophages (TAMs) are the most critical cell population. Broadly speaking, TAMs include tissue-resident macrophages (microglia, MG), bone marrow-derived monocytes (BMDMs), polarized macrophages (M1/M2), and dendritic cells (DCs) (6). Generally, TAMs usually refer to inhibitory polarized macrophages (M2) or tumor-infiltrating myeloid suppressive cells, which are the main culprits of immunosuppression in the TME (7). They can enhance the stemness, proliferation, survival, and migration abilities of cancer stem cells (GSCs) while suppressing adaptive immune responses and having extensive symbiotic relationships with tumor cells. Several drugs targeting TAMs have been developed, including CSF-1R inhibitors, CCL2/CCR2 blockers, and CD47-SIRPα modulators (8). These drugs have shown some promise in other types of cancer, but the complexity and diversity of TAMs in the immune microenvironment of glioma make the effectiveness of these treatment methods less satisfactory.

Chlorogenic acid (CHA), also known as 5-O-caffeoylquinic acid, is a natural phenolic acid produced by plants such as tea leaves and coffee beans, and its safety has been recognized (9, 10). CHA has been shown to reduce the risk of various diseases with a wide range of biological activities, such as antimicrobial, immunomodulatory, antioxidant, and anticancer properties (11, 12). CHA modulates the cell cycle through multiple mechanisms or promotes cell death through other pathways, exerting cytotoxic effects (13–15). Recent research has revealed that CHA can induce differentiation in various cancer cells (including glioma cells) and reduce the malignancy of tumor cells, with a therapeutic effect in animal experiments comparable to that of temozolomide (10). CHA also shows a positive effect on improving the immunosuppressive nature of glioma TME.

Our laboratory has been dedicated to studying the anti-glioma effects of CHA. Our early research has indicated that CHA can activate the JAK-STAT pathway, inducing macrophage polarization into the M1 phenotype rather than the M2 phenotype, thereby exerting anti-tumor effects (16, 17). Recent studies have demonstrated that CHA downregulates PD-L1 expression through the STAT1-IRF1 pathway, promotes the infiltration and activation of memory T cells and effector T cells surrounding tumor tissues, and enhances the efficacy of PD-1 antibodies (18, 19). CHA has now entered the clinical trial phase. According to the research results on the efficacy and safety of CHA in treating high-grade gliomas (CTR20160113), CHA has good safety and provides preliminary clinical benefits for high-grade glioma patients who relapse after receiving standard therapy (20).

Nevertheless, our understanding of the mechanisms of CHA in treating gliomas, especially its targeting effects on the immune microenvironment, is still limited. In this study, the development and differentiation trajectories of tumor-infiltrating myeloid cells and microglia were re-examined, and an in-depth investigation of the effects of CHA on the immune microenvironment of gliomas was conducted. The results of this study may provide new insights into TAMs and TME regulation. Meanwhile, the clinical data on the treatment of recurrent glioma patients with CHA were reported for the first time, serving as an important basis for the therapeutic effects of CHA.

## 2 Materials and methods

### 2.1 Anti-cancer drugs

CHA was obtained from the Jiuzhang Biochemical Engineering Science and Technology Development Co., Ltd. (Chengdu, Sichuan, China) with a purity of more than 99%. CHA was dissolved in normal saline (NS) at a concentration of 100 mM as a stock solution for the *in-vivo* experiment.

### 2.2 Orthotopic transplantation model in C57BL6/N mice

Female C57BL6/N mice (age 6 weeks, 15-20g) were selected to investigate the *in vivo* anti-glioma effects of CHA. The GL261-Luc cells labeled with luciferase were used to establish the orthotopic glioma transplantation model, and the anti-tumor effect was examined using an *in-vivo* imaging system. The GL261-Luc cell line was digested with trypsin, resuspended in PBS solution for density adjustment to 1.5×10^5/5 μL, and kept on ice for further use. Immunocompetent C57BL6/N mice were anesthetized through intraperitoneal injection of tribromoethanol, and the ear bar was inserted into the external auditory meatus of the mouse to adjust the position of the incisor bar of the positioning device to 3.3 ± 0.4mm below the horizontal line, ensuring that the mouse skull was in a horizontal position. Subsequently, surgery was performed on the skin of the mouse’s head. Under the guidance of a stereotactic apparatus, with the anterior fontanelle as the origin, the needle was inserted vertically 3mm at the position 2mm behind the anterior fontanelle and 2mm lateral to the midline. Before injection, the needle was slightly pulled back about 0.5 mm, and the GL261-Luc single-cell suspension was injected at a speed of 2.5μL/min through a micro pump. After injection, the needle was kept in place for 1 minute and then retracted. The puncture hole was sealed with bone wax, and the scalp wound was finally disinfected with iodine and sutured.

Three days after inoculation, the surviving mice were photographed in an *in-vivo* imaging system and randomly allocated into the experimental group and control group (6 mice per group). The experimental group was intraperitoneally injected with CHA (40mg/kg) every day, and the control group was intraperitoneally injected with physiological saline (40mg/kg). This dose was set referring to a previous study (16). On the 7^th^, 14^th^, and 21^st^ days after inoculation, tumor growth was recorded in an *in-vivo* imaging system, and the survival days of mice in the control and experimental groups were recorded. The bioluminescence values were calculated to evaluate the tumor inhibition effect of CHA. Meanwhile, the activity and mental condition of mice were observed following the ethical requirements for experimental animals. We considered removing an animal from the experiment and performing euthanasia under the following circumstances: body temperature exceeding the normal range by more than 4°C for 12 consecutive hours; anorexia and dehydration for more than 3 days; wound infection or tumor rupture; signs of lethargy, aggression, panic, escape attempts, or other abnormal behaviors. If any of these conditions occur, euthanasia should be carried out promptly.

### 2.3 Tumor tissue dissociation

The brain tissues of mice in the experimental group (n = 3) and control group (n = 3) were extracted, with their tumor and peritumoral tissues excised and fully immersed in tissue preservation fluid for temporary storage. Within the following 24 hours, samples were subjected to uniform dissociation to generate single-cell suspension samples. Tumor tissues were washed in a 50-mL centrifuge tube by adding 10 mL chilled 1X DPBS. After that, tumor tissues were placed in a petri dish and sectioned into small pieces (about 2-4 mm^3^). Enzyme mix was prepared in a gentleMACS™ C Tube by adding 2.35 mL RPMI 1640 or DMEM, 100 µL Enzyme D, 50 µL Enzyme R, and 12.5 µL Enzyme A (21). Tumor tissue sections were transferred to the enzyme mix-contained C Tube. The C Tube was tightly closed and attached upside down to a sleeve of a gentleMACS Octo Dissociator with Heaters. The program 37C_m_TDK_1 was run. At the end of the run, the C Tube was detached from the Dissociator. Centrifugation was performed at 300 rcf for 30 sec at room temperature. The supernatant was removed without disturbing the cell pellet. Next, 10 mL RPMI 1640 or DMEM was added and gently pipette mixed to resuspend the cell pellet. The cell suspension was filtered through a prewetted 70-µm MACS^®^ SmartStrainer placed on a 50-mL centrifuge tube. The strainer was washed with 10 mL RPMI 1640 or DMEM and the wash in the tube was collected with the cell suspension. The cell suspension was centrifuged at 300 rcf for 7 min at room temperature. The supernatant was removed without disturbing the cell pellet. Afterward, 1 mL chilled 1X Red Blood Cell Removal Solution was added to the cell pellet and gently pipette mixed to resuspend the cells. After incubation for 10 min at 4°C, 10 mL chilled Wash Buffer was added, followed by centrifugation at 4°C at 300 rcf for 10 min. The supernatant was removed without disturbing the cell pellet, and then 5 mL chilled Wash Buffer was added and gently pipette mixed to resuspend the cell pellet. The cell concentration was determined using a Countess^®^ II FL Automated Cell Counter or hemocytometer. An appropriate volume of chilled wash buffer was added to the cell suspension and gently pipette mixed to achieve the target cell concentration of 700–1200 cells/µL (7x10^5^-1.2x10^6^ cells/mL). Proceed immediately with the 10x Genomics^®^ Single Cell protocol.

### 2.4 Single-cell RNA sequencing

The protoplast suspension was loaded into Chromium microfluidic chips with 30 chemistry and barcoded with a 10× Chromium Controller (10X Genomics) (22). RNA from the barcoded cells was subsequently reverse-transcribed and sequencing libraries were constructed with reagents from a Chromium Single Cell 30 reagent kit as per the manufacturer’s instructions. Sequencing was performed with Illumina (HiSeq PE125) following the manufacturer’s protocol.

### 2.5 scRNA-seq data preprocessing and normalization

Raw sequencing data were demultiplexed and converted into fastq format using CellRanger v3.0.1 (10× Genomics) and Illumina (22, 23). The sequencing results were mapped to the mouse genome GRCm38 (mm10) obtained from the 10× Genomics website and quantified using CellRanger. A total of 35,359 cells were identified by CellRanger. Data analysis was performed using Seurat (v4.4.0) in R (24). Unless otherwise specified in our methods, all other quantitative parameters were set to default values. Possible empty droplets, low-quality cells (cell-level filtering), and possible multiple droplets were removed with the following thresholds: 1. cells with transcripts less than 200 or more than 3000; 2. UMI counting greater than 500 per cell; 3. more than 500 genes detected per cell; 4. mitochondrial count percentage less than 0.2; 5. a complexity greater than 0.80 to exclude low-complexity cell type contamination according to the calculation of the complexity using log10GenesPerUMI. Additionally, gene-level filtering (cell-level filtering) was performed, where genes with zero expression in all cells were removed, and only those expressed in 10 or more cells were retained. After the above filtering, a total of 25,334 cells in the dataset proceeded to the next steps of the process.

Harmony integration (25) and differential normalization were performed on each sample, and unwanted variations were regressed out such as mitochondrial genes, ribosomal genes, and cell cycle genes. The gene expression measurements for each cell were normalized by multiplying the total number of transcripts within the cell by the default scaling factor, and the normalized values were log-transformed (using the “LogNormalize” method). Following the Seurat workflow, variance-stabilizing transformation (“vst”) was used to identify the 2000 most variable genes across each replicate. Principal component analysis (PCA) was employed for dimensionality reduction of the data. A nearest-neighbor graph was constructed using the neighbor function, and an unsupervised clustering algorithm was applied using the Louvain function (resolution = 0.4). The dimensionality reduction results were projected onto a two-dimensional plane using UMAP.

### 2.6 Cell annotation and differential gene analysis

After identifying immune cells, monocyte-macrophages, microglia, T cells, NK cells, and B cells based on the report/canonical scRNA markers of different types of cells (**Supplementary Table S1**), we repeated the aforementioned clustering steps for further study. The Wilcoxon rank-sum test provided by Seurat was used to study the characteristic upregulated/downregulated genes between different cell subpopulations and groups. GSEA analysis was further performed on these genes. The GSEA analysis was conducted in the R package clusterProfiler (v4.6.2) (26). We used the Gene Ontology: Biological Process (GO: BP) and Reactome databases built into clusterProfiler to analyze the biological characteristics of differential genes (27). GO annotation for mice was performed using the org.Mm.eg.db (v3.4.0) package (28). Redundant results were also eliminated using the simplify function (cutoff = 0.7).

### 2.7 Pseudotime analysis with monocle3

Monocle 3 (v3.1.4) is a tool for scRNA-seq data analysis that can identify transitions and trajectories of cellular states (29). PCA was used for dimensionality reduction before pseudotime analysis, with the PC values consistent with the parameters used for dimensionality reduction clustering in Seurat. Unless otherwise specified in our methods, all other quantitative parameters were set to default. The parameters for Monocle3 were all set to their default values. In identifying the starting point of pseudotime (learn_graph), we manually delineated based on the biological significance of the cell subpopulations and the minimum value of the ‘pseudotime’ parameter, and the results were returned to Seurat for further analysis.

### 2.8 Gene set variation analysis

GSVA is a non-parametric unsupervised algorithm that is centered on gene sets and can transform a gene expression matrix into an expression matrix based on gene sets. The similarity between the two groups of differential genes (CHA-Deg and Act-Deg) was compared using the R package GSVA (30).

### 2.9 Network pharmacology-related targets

The chemical structure of CHA (Canonical SMILES) was obtained through PubChem (https://pubchem.ncbi.nlm.nih.gov/) (31). The SMILES results were then imported into the SwissTargetPrediction database (http://www.swisstargetprediction.ch/) (32) and the SuperPred database (https://prediction.chlorogenicacid.rite.de/) (33) to predict compound targets, and the results from both databases were integrated. We used the keywords “glioma” and “microglia activation” to obtain network pharmacology targets related to the disease and microglia in the OMIM database (http://www.omim.org/) (34) and the GeneCards database (http://www.genecards.org/) (35), respectively. The results were integrated from these two databases.

### 2.10 Construction of protein-protein interaction networks and topological analysis

Candidate targets were imported into the STRING (v12.0) (https://string-db.org/) to investigate the role of microglia in the glioma treatment process with CHA. A PPI network was constructed and subsequently visualized using Cytoscape (v3.10.1). To obtain topological parameters, we calculated the clustering coefficient, node degree distribution, shortest path, and length distribution using the network analyzer settings. The network was analyzed using “Analyze Network” in Cytoscape. The size and color of each node reflected the degree of influence on the comprehensive score, thereby obtaining the final PPI network. Additionally, cluster analysis was performed on the PPI network using 11 algorithms based on the Cytohubba (36) (including MCC, DMNC, MNC, Degree, EPC, BottleNeck, Eccentricity, and Closeness) to extract core gene modules.

The 3D structural files of CHA and the core target STAT1 (pdb code: 1yvl) were obtained from the TCMSP database (https://old.tcmsp-e.com/tcmsp.php) (36) and the Protein Data Bank database (https://www.rcsb.org) (37), respectively, to further study the spatial conformation of the binding between CHA and its core targets. Molecular docking was performed using Discovery Studio (https://www.3ds.com/products/biovia/discovery-studio) to obtain the binding energy and visualize the docking situation. After manually removing water, the “Prepare Protein” function was used to automatically add hydrogens, calculate charges, and select the protein as the receptor. The size of the site sphere was set to -24.0122, 0.521902, 108.156, and 11.7039. Docking was performed using the CDOCKER functionality. A binding energy below zero implied that the ligand was capable of freely associating with the receptor; a binding energy below -5 kcal/mol denoted that the ligand exhibited strong binding activity with the target protein; a binding energy value below -7 kcal/mol signified an exceptionally high affinity of the ligand for the target protein.

## 3 Results

### 3.1 The tumor suppression effect of CHA on the transplantation model

**Figure 1:**
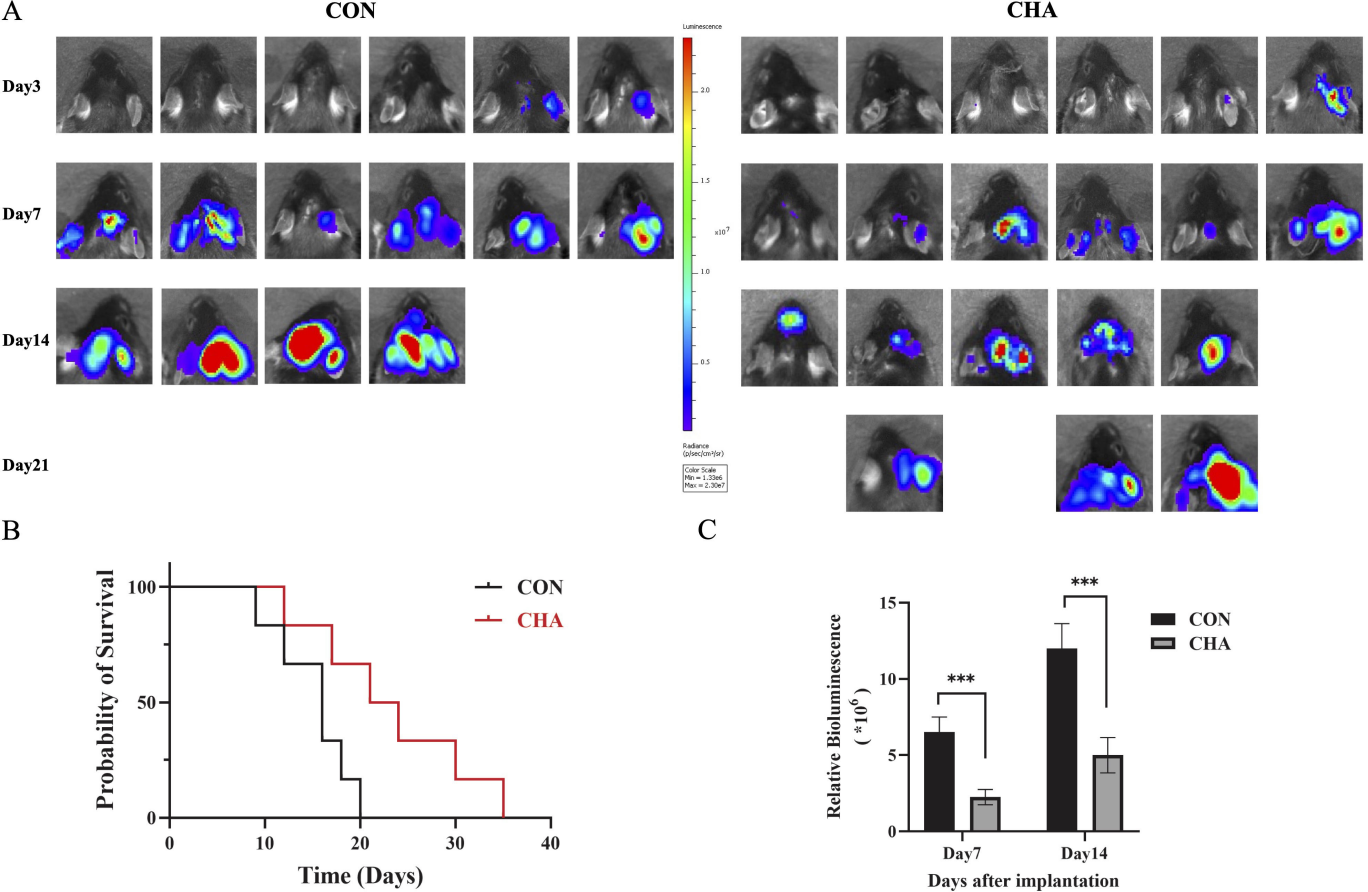
Effect of CHA on GL261-Luc tumor model in immunized normal mice (C57BL6/N). **(A)** Biological *in vivo* imaging of mice in the experimental and control groups on days 3, 7, 14, and 21 of inoculation. **(B)** Survival curves of mice in the experimental and control groups. **(C)** Tumor size statistics of mice in the experimental and control groups on days 7 and 14 (*p* < 0.05), ***: p<0.001.

To investigate the effects of CHA on the immune microenvironment of glioma in patients, we selected mice of the C57BL6/N strain to construct an orthotopic GL261 glioma model, which more closely simulates the immune microenvironment of human glioma. Tumor growth in the mouse model was quantitatively analyzed and evaluated using small animal *in vivo* imaging technology. The experimental group treated with CHA showed a significant reduction in luminescence intensity, which represents tumor size, compared to the control group (*p* < 0.001) (**Figure 1A**). Mice treated with saline injections in the control group exhibited a significant increase in tumor volume by day 14 compared to day 7, and all mice in the control group died within 21 days of the experiment. In contrast, the CHA-treated group showed a significant survival advantage: more than half of the mice were still alive by day 14, and half were able to survive until day 21 (**Figures 1B, C**). These results suggested that CHA not only significantly inhibited the growth of orthotopically transplanted glioma in mice but also improved the survival rate of glioma mice.

### 3.2 Single-cell atlas of GL261 glioma-microenvironment under the influence of CHA

scRNA-seq analysis was performed on mixed tumor tissues from three experimental mice with CHA treatment for 14 days and three control mice to investigate the impact of CHA on the immune microenvironment of mouse glioma. After quality control, a total of 25,334 cells were analyzed, and 18,634 expressed genes were detected (**Supplementary Figure S1**, **Supplementary Table S1**). Unsupervised clustering analysis was used to categorize the cells into 19 clusters and the high-dimensional data were displayed on a two-dimensional plane using UMAP (**Supplementary Figures S2A, B**). Among the annotated 22,262 immune cells (Ptprc^+^), a substantial number of infiltrating T or NK cells (Cd2^hi^, Cd3d^hi^, Cd3e^hi^) were identified. These immune cells also included a significant number of TAMs (Tgfbi^hi^, Ifitm2^hi^, Ifitm3^hi^) and a small number of B cells (Cd79a^hi^, Cd19^hi^, Ms4a1^hi^) (**Supplementary Figures S2C, D**). We distinguished the TAMs population into central microglia and bone marrow-derived populations using CD49a (Itga4), identified microglia using classical markers (Tmem119, Crybb1, and P2ry12), preliminarily identified the Mo/Mφ fraction using monocyte markers (Chil3 and Plac8) and macrophage markers (Ifitm2, S100a6, and S100a11), and identified DCs using Cd24a and Ly75 (**Supplementary Figures S2E, F**) (37–39).

**Figure 2:**
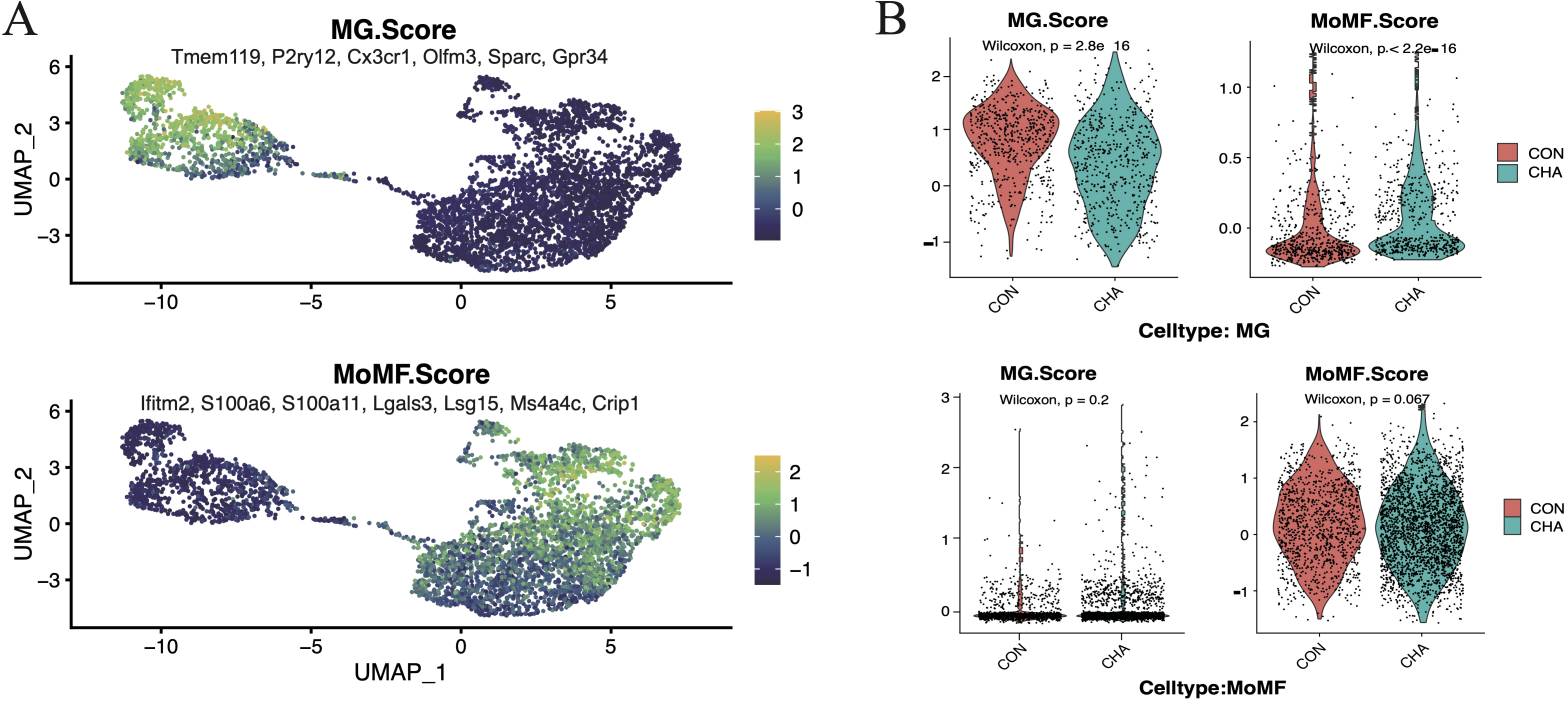
Effect of CHA on microglia and macrophage characteristics. **(A)** Microglia feature scores and macrophage feature scores in innate immune cells. **(B)** Violin plots demonstrating the two scores for the experimental and control groups in mono-macrophages and microglia, respectively.

MG scores and MoMF(Mono-macrophages) scores designed by the average expression levels of specific genes were employed to further investigate the influence of CHA on the two main populations of TAMs (**Figures 2A, B**, **Supplementary Table S1**). Our analysis results showed that these scores effectively differentiated the two cell populations. After treatment with CHA, the MG score of the microglia population was decreased, while the MoMF score was notably increased (*p* < 0.05). This indicated that microglia may have undergone a shift toward macrophage characteristics. In contrast, the macrophage population maintained a low MG score after CHA treatment, but the MoMF score was not significantly changed (*p* > 0.05), which reflected that the action mechanism of CHA on the macrophage population was different from that on microglia and involved a more complex regulatory process.

### 3.3 Reconstruction of the differentiation trajectory of BMDMs

**Figure 3:**
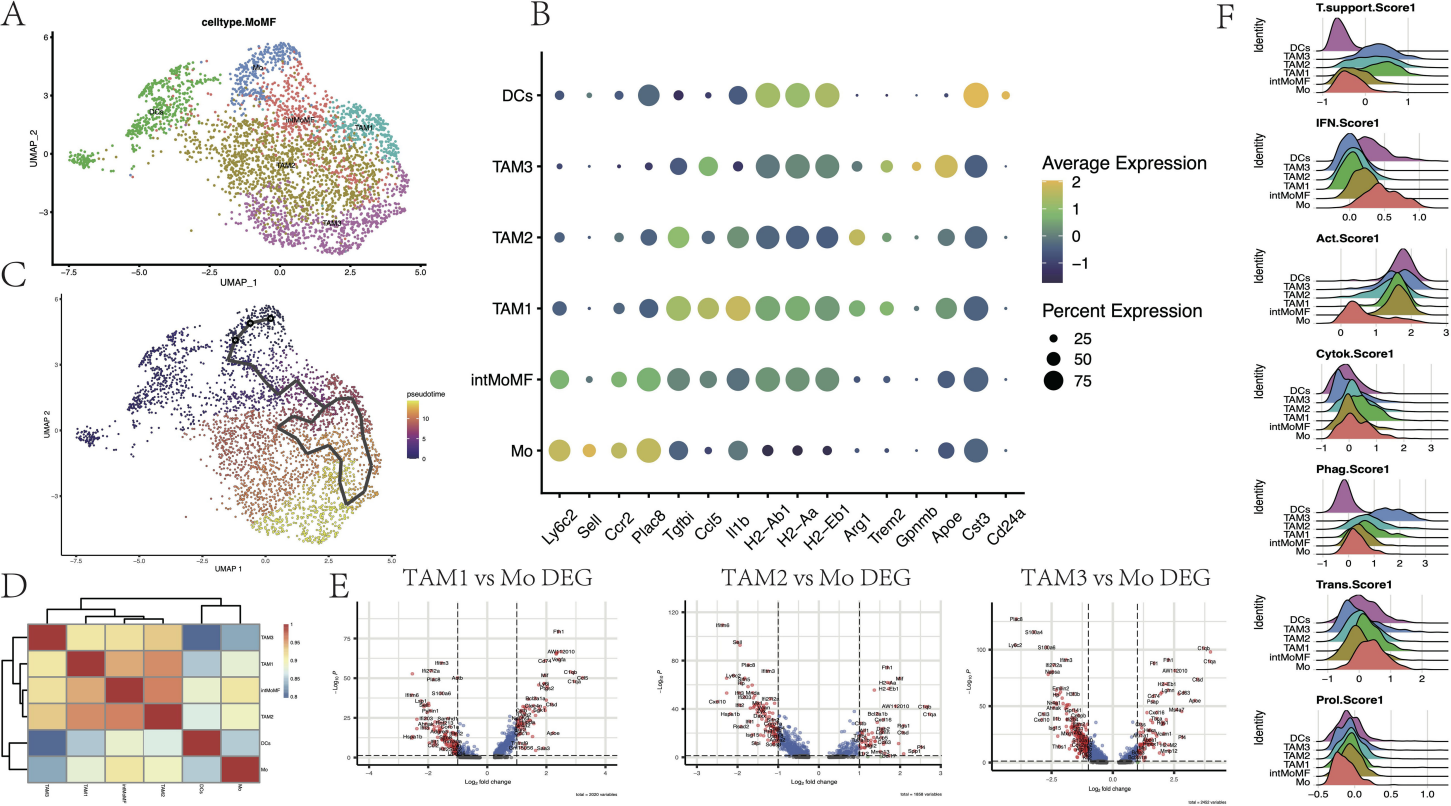
Subpopulation heterogeneity of monocyte-macrophages. **(A)** UMAP plot demonstrating single-cell mapping of monocyte-derived subpopulations. **(B)** Markers of monocyte-derived subpopulations. **(C)** Pseudotime differentiation trajectory of monocyte-macrophages. **(D)** Heatmap demonstrating Spearman’s correlation between different cell subpopulations. **(E)** Differential genes of TAM1, TAM2, and TAM3 subpopulations compared to monocyte Mo (*p* < 0.05). **(F)** Ridge plot indicating the degree of tumor-supporting, activation of the interferon (IFN) pathway, tumor activation, cytokine secretion, phagocytosis, expression of transcription factors, and cell proliferation of different cellular subpopulations (see **Supplementary Table S1** for scoring genes).

TAMs are a highly heterogeneous group of cells in the human glioma microenvironment. BMDMs infiltrate from the bloodstream into the tumor tissues, becoming a part of TAMs, and differentiate into phenotypes with different functions under the stimulation of specific signals in glioma. In scRNA-seq, the BMDMs were considered as a continuum, and six subgroups within this population were identified (**Figures 3A, B**). We determined monocytes (Ly6c2^hi^, Sell^hi^, Ccr2^hi^, Plac8^hi^, and Tgfbi^low^), to the transitional state intMoMF (Ly6c2^hi^ and Tgfbi^hi^), and then to functionally differentiated macrophages TAM1 (Il1b^hi^ and MHC-II^hi^), TAM2 (Arg1^hi^), and TAM3 (Gpnmb^hi^ and Apoe^hi^), as well as DCs (Cst3^+^, Cd24a^+^, and MHC-II^hi^).

Through pseudotime analysis, the differentiation trajectory of the BMDMs in the glioma microenvironment was depicted (**Figure 3C**, **Supplementary Figure S3**). High expression of Cd83, Chil3, and Plac8 in Mo was the earliest time marker, accompanied by the activation of the inflammatory response IFN pathway (Ifitm6 and Ifitm2). Over time, the expression of these inflammatory genes was rapidly declined, while the expression of factors that promoted tumor growth (such as Vegfa and Arg1 that promoted tumor angiogenesis, and the signature genes Trem2 and Apoe that promoted tumor growth) continued to rise. Eventually, the expression of Gpnmb and Il18bp was elevated, indicating that the ‘hijacking’ of the TAM population by glioma cells was complete. These results were consistent with the research conclusions of Daniel et al. (39).

Furthermore, Spearman correlation analysis was performed to explore the correlation between gene expressions of various cell subgroups, which were presented through a heatmap (**Figure 3D**). It was observed that Mo maintained a certain correlation with both DCs and TAMs on either side, while there was a stronger correlation among the internal subgroups of macrophages, which was consistent with pseudotime analysis results. Among the three subgroups of TAMs, these cells had higher scores for tumor activation (MHC-II and Cd52) and phagocytic function (Apoe and Lgals5), indicating that these TAMs still had certain antigen presentation capabilities (**Figures 3E, F**, **Supplementary Table S1**). Compared to Mo, the expression of complement-related genes (C1qa and C1qb) and lysosomal cathepsins in TAMs was increased (Ctss and Ctsd). In addition to the decline in the expression of IFN pathway-related genes (Il1b, Ifitm2, Ifitm3, and Ifi204) and monocyte marker genes (Ly6c2 and Plac8), the expression of genes encoding calcium-binding proteins (S100a4, S100a6, and S100a10) and the gene encoding chemokine Cxcl10 was also declined (*p* < 0.05, log2FC=1). Among them, TAM1 showed a relatively strong polarization trend toward the M1 type, while TAM3 had a more comprehensive expression of immunosuppressive genes (**Supplementary Figure S3**). These results help us explore the effects of CHA on TAMs and provide a new perspective for understanding the complexity of the glioma microenvironment.

### 3.4 The regulatory effect of CHA on the transcriptional characteristics of BMDMs

**Figure 4:**
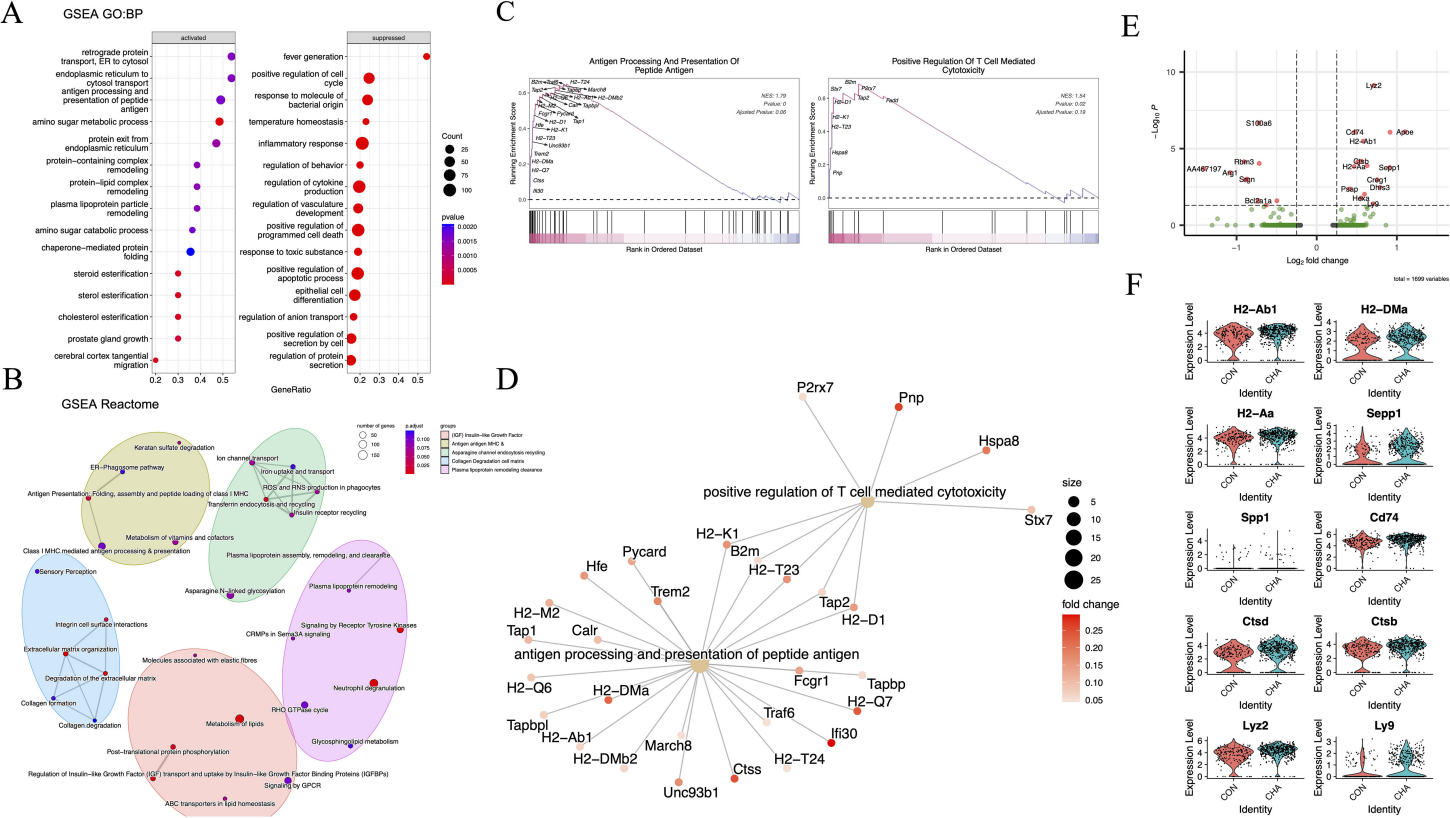
The functional effects of CHA on monocyte-macrophages. **(A)** Bubble diagram showing the results of GO: BP analysis. **(B)** Network diagram showing the results of Reactome analysis. **(C, D)** GSEA plots and gene networks showing the GO: BP term: positive regulation of T cell-mediated cytotoxicity and antigen processing and presentation of peptide antigen. **(E)** Volcano plot demonstrating the differentially expressed genes in the TAM3 subpopulation in the experimental group compared to that in the control group (log2FC > 0.25, *p* < 0.05). **(F)** Violin plot demonstrating differentially upregulated genes in TAM3 in the experimental group.

Our laboratory previously explored the regulatory effect of CHA on macrophages using animal models and discovered the potential of CHA to re-polarize M2 phenotype to the M1 (16). Subsequently, GSEA was employed to explore the activated gene networks after the action of CHA to further investigate the regulatory effect of CHA on TAMs and the underlying mechanisms. The differential genes were analyzed through GO: BP and Reactome pathway databases (**Figures 4A, B**, **Supplementary Table S4**).

Our results showed that multiple biological pathways highly related to adaptive immune modulation were upregulated in the CHA treatment group compared to those in the control group. The significantly upregulated pathways were those directly related to adaptive immunity, such as antigen processing and presentation of peptide antigen (GO:0048002), retrograde protein transport, ER to cytosol (GO:0030970), endoplasmic reticulum to cytosol transport (GO:1903513), protein exit from endoplasmic reticulum (GO:0032527), as well as several pathways related to amino sugar and lipid metabolism (GO:0006040, GO:0034433, GO:0034434, and GO:0034435) (**Figures 4A, D**). Additionally, the correlation between differential genes and adaptive immunity was assessed. As indicated by GSEA analysis results, genes such as MHC-II (H2-T23, H2-D1, and H2-Aa), Pnp, and Hspa8 were involved in T cell immunity (GO:0001916) (*p* < 0.05) (**Figure 4C**). Reactome pathway analysis was then performed on these differential genes, and it was found that the enriched pathways included the IGF signaling pathway (R-MMU-381426), antigen presentation (R-MMU-983170), and lipid metabolism involved in the regulation of immunity (R-MMU-6798695) (**Figure 4B**). In the comparison of differential genes in the TAM3 subgroup (*p* < 0.05, log2FC > 0.25), we observed significant upregulation of gene expression for MHC-II (H2-Ab1, H2-DMa, H2-Aa, and Cd74), osteopontin (Spp1), and lysosomal proteins (Cstb and Cstd) (*p* < 0.05) (**Figures 4E, F**). The upregulation of these molecules may reflect changes in the activation state of macrophages. They collectively participate in the immune system’s surveillance and clearance of tumor cells by enhancing antigen presentation, regulating adaptive immune responses, and participating in extracellular matrix remodeling, providing new strategies and insights into molecular mechanisms for immunotherapy of glioma.

### 3.5 The anti-tumor activation trajectory of microglia

**Figure 5:**
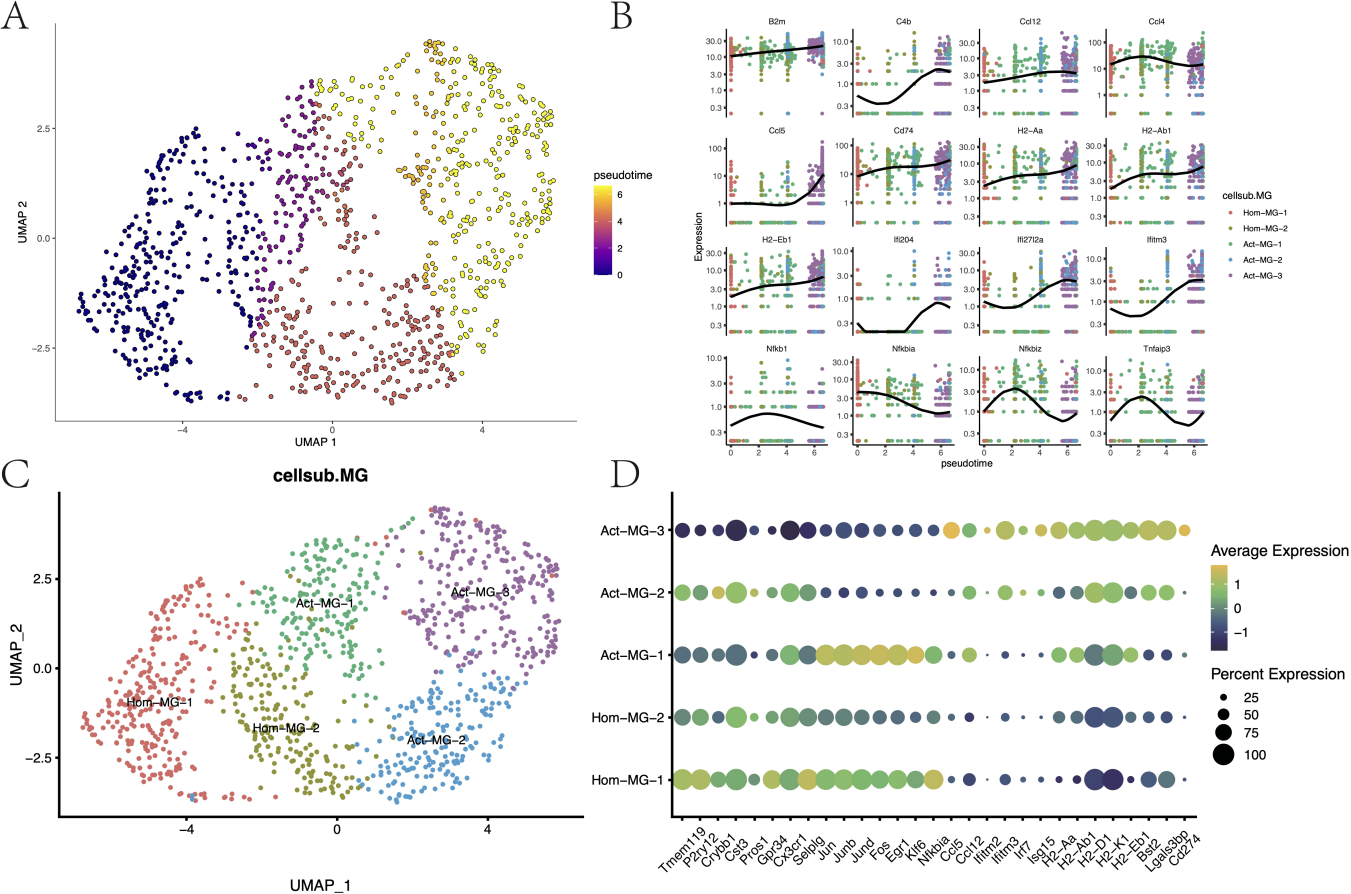
Differentiation trajectories and functional characteristics of microglia. **(A)** Pseudotime differentiation trajectories of microglia. **(B)** Trajectory node genes for microglia activation. **(C)** Subpopulation mapping of microglia. **(D)** Expression of marker genes in microglia subpopulations.

Based on the expression patterns of the microglial marker gene Tmem119 and MHC-II-related genes, microglia were allocated into two major subgroups: the homeostatic microglia Hom-MG (Tmem119^hi^ and MHC-II^low^) and the activated microglia Act-MG (Tmem119^+^ and MHC-II^hi^). The anti-tumor response process of microglia under glioma activation was reconstructed, which showed a series of gene changes involved in the activation process of microglia (**Figures 5A–C**, **Supplementary Figure S4A**): including the enhancement of MHC-II gene expression (H2-Aa, H2-Ab1, H2-Eb1, Cd74, and B2m), activation of the IFN pathway (Ifitm3, Ifi27I2a, Ifit2, and Ifit3), activation of the complement system (C4b), and the weakening of inhibitors of the NFkB pathway (Nfkb1, Nfkbia, Nfkbiz, and Tnfaip3).

Through differential expression analysis (*p* < 0.05, log2FC > 0.25), the transcriptional characteristics of these subgroups were further delineated (**Supplementary Figure S4C**; **Figure 5D**). The Hom-MG-1 subgroup highly expressed the innate characteristic genes of microglia (Tmem119, P2ry12, Crybb1, Cst1, Gpr34, and Cx3cr1), poorly expressed tumor activation-related genes, and expressed early activation genes to a certain extent (Jun, Junb, Jund, and Fos). These suggested that these cells were homeostatic microglia that have not yet been activated by the tumor but have the potential to be activated. The Hom-MG-2 cluster showed a mixed expression pattern of homeostatic genes, transcription factors, and tumor activation genes, indicating that these cells were in a state of dynamic change. Act-MG-1, based on expressing MHC-II, remarkably upregulated a series of transcription factors and cytokines (Ccl3, Ccl4, Jun, Junb, and Jund), indicating that this cluster of cells was a type of active microglia that can quickly respond to tumor stimulation. The Act-MG-3 subgroup, however, showed high-activity cells activated by the tumor, with strong phagocytic/lipid metabolic activity (Cst7 and Apoe), but the expression of transcription factors and cytokines was weakened, indicating that these cells had been reprogrammed into polarized functional cells. It’s worth noting that PD-L1 (Cd274) expression in the Act-MG-3 subgroup was upregulated, indicating that activated microglia were likely to have certain immunosuppressive properties, which is consistent with previous reports. In contrast, Act-MG-2 did not have such immunosuppressive characteristics but showed a more comprehensive transcriptional profile, indicating that these cells were in a transitional stage, similar to the Hom-MG-2 subgroup, but the activation degree of MG-Act-2 seemed to be higher (**Supplementary Figure S4B**, **Supplementary Table S1**). These results reflected the process by which microglia become more “macrophage-like,” revealing the complex dynamics of microglia in the glioma microenvironment.

### 3.6 Effects of CHA on the activation of microglia

**Figure 6:**
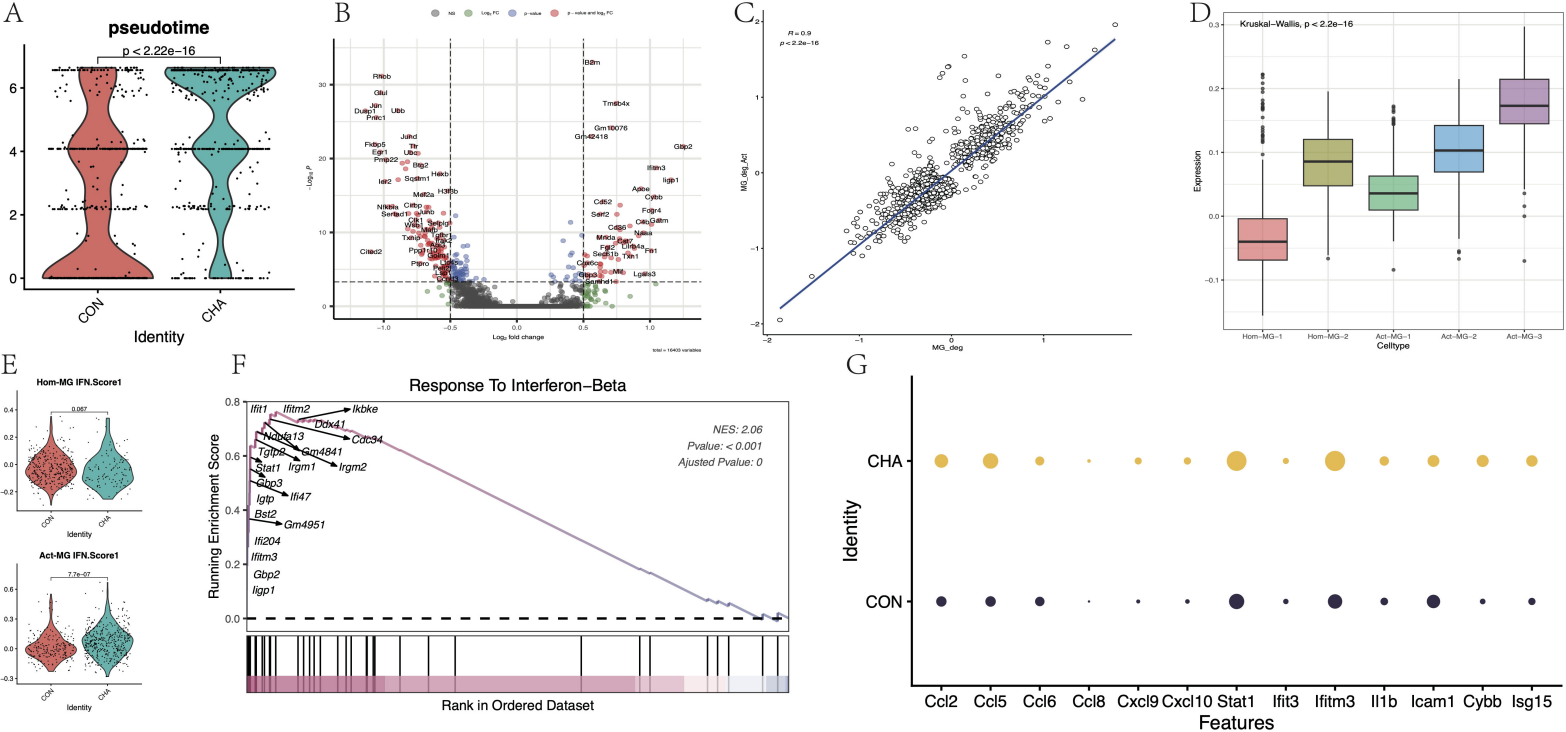
Effect of CHA on microglia activation status. **(A)** Comparison of the difference in proposed chronological scores between the CHA experimental and control groups (*p* < 0.05). **(B)** Microglia differentially expressed genes in the experimental group compared to the control group. **(C)** Comparison of the differentially expressed genes (CHA-Deg) and the differentially expressed genes between microglia subpopulations of Act-MG and Hom-MG subpopulations of microglia between the groups (Act-Deg). Correlation between the two Deg genes. **(D)** GSVA analysis examined the degree of similarity between the differential genes in the two groups. **(E)** Comparison of the average expression of IFN pathway genes between the two populations Hom-Act and Act-MG, in the experimental and control groups. **(F)** GSEA plot in response to the IFN-b pathway. **(G)** Expression of genes downstream of chemokines and JAK-STAT.

Whether CHA was involved in the activation process of microglia was further investigated. In the microglia of mice from the experimental group with CHA treatment, the “pseudotime scores” (calculated by monocle3) were significantly higher than those of the control group, suggesting that CHA can promote the transition of microglia to an activated state (**Figure 6A**). Differential analysis was performed on the microglia from both the experimental and control groups, and upregulated and downregulated gene sets were obtained (MG_CHA_Deg) (**Figure 6B**, **Supplementary Table S3**). Meanwhile, the differential expression between the two subgroups of Act-MG and Hom-MG cells was also analyzed, and the gene set ActMG-Deg was obtained. By calculating the Pearson correlation coefficient between these two sets of differentially expressed genes (**Figure 6C**), it was found that the changes in gene expression were highly synchronized (R = 0.9, *p* < 2.2e-16). GSVA was used to further corroborate this hypothesis, which analyzed five different microglial subgroups and assessed the expression of the CHA-Deg gene set in these subgroups (**Figure 6D**). According to GSVA results, subgroup Act-MG-3 significantly enriched the expression of MG_CHA_Deg, while subgroup Hom-MG-1 had the lowest enrichment of MG_CHA_Deg (*p* < 2.2e-16), which is consistent with our hypothesis that CHA can promote the activation process of microglia.

GSEA analysis was conducted on the CHA-Deg differential gene set and enriched multiple upregulated or downregulated GO: BP terms (**Supplementary Figure S5A, B**, **Supplementary Table S4**). Among the upregulated pathways, the IFN regulation pathway (GO:0035456) was noticeably enriched with the highest normalized enrichment score (NES = 2.06). Additionally, this differential gene set also significantly enriched other pathways, such as innate immune response (GO:0045087), complement pathway activation (GO:0006956), and monocyte migration (GO:0071675, GO:0071674). These upregulated pathways reflected that microglia showed signs of activity and played an important role in immune regulation, metabolic control, and pathogen response. Most of the immune-related activation genes (Ccl2, Ccl5, 6, 7, 8, Ccl24, Irgm1, 2, Gbp2, 3, 5, S100a8, and S100a9) were directly involved in the production and signaling of IFN. The upregulation of Stat1 further emphasized the importance of the JAK-STAT signaling pathway in this process. Furthermore, we focused on the differences in the activation degree of the IFN pathway between the Hom-MG and Act-MG cell populations (**Figure 6E**). The results revealed that CHA strengthened the response to the IFN pathway more significantly in the Act-MG population (*p* < 0.05). Several important downstream genes in this pathway were assessed (**Figures 6F, G**), and all these showed enhanced expression, further emphasizing that CHA regulated the immune response of microglia through the JAK-STAT pathway.

### 3.7 Cluster analysis based on PPI network, core target prediction, and molecular docking

**Figure 7:**
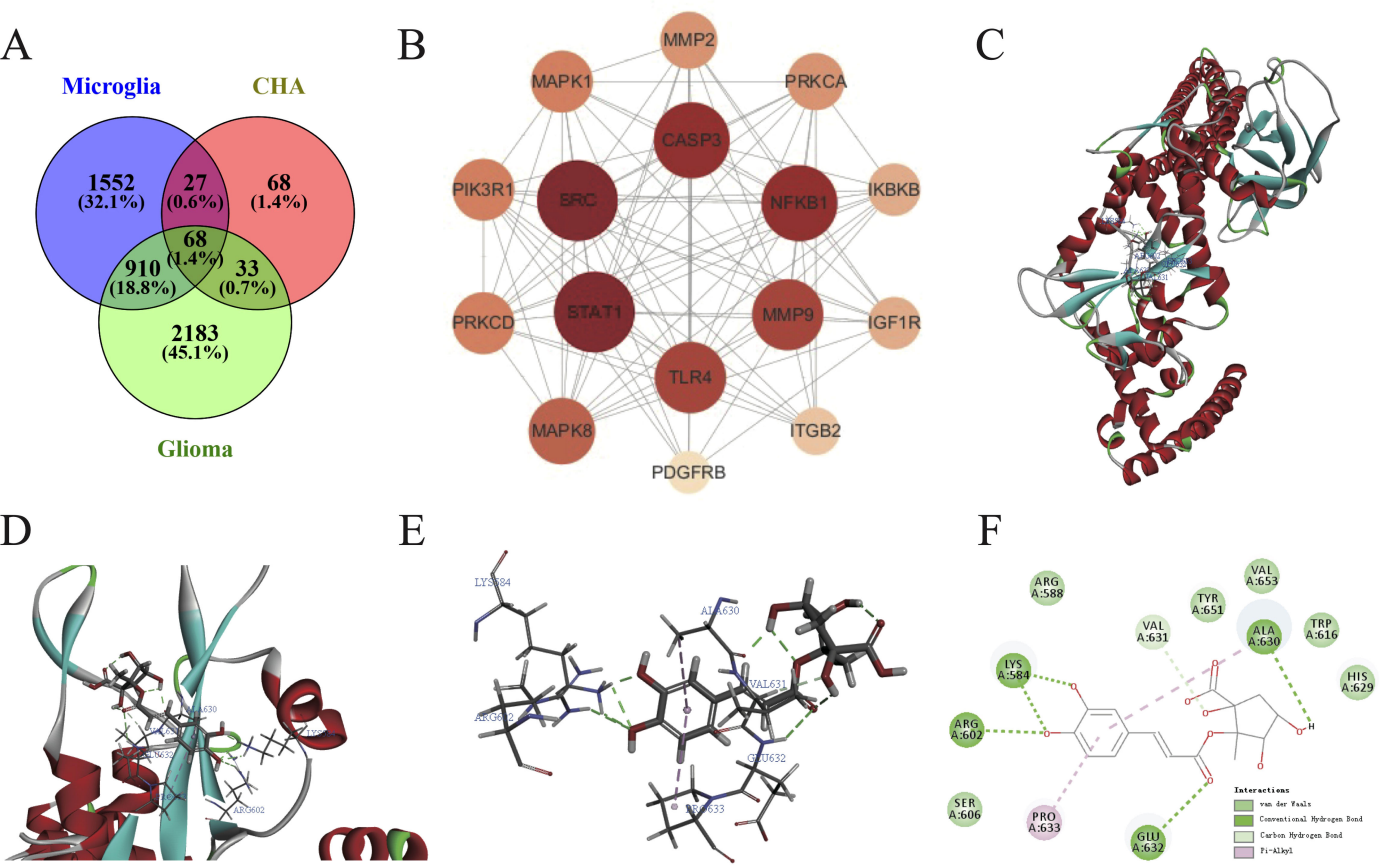
Construction of PPI network and molecular docking of CHA in regulating microglia function. **(A)** Common targets of microglia activation, CHA, and glioma. **(B)** Algorithmically optimized protein-interacting core genes. **(C–E)** 3D conformation showing the binding site of CHA to STAT1 (marked in red), with the lowest binding energy of -41.761 KJ/mol. **(F)** 2D diagram showing the forces formed in docking. Van der Waals forces, hydrogen bonds, and π-bonds in the binding site of CHA and STAT1 are marked in light green, green, and pink, respectively.

The SwissTargetPrediction and Supred databases were utilized to predict the pharmacological targets of CHA, and a total of 196 potential targets were identified. Subsequently, 3194 targets associated with glioma and 2558 targets related to microglial polarization were collected from the OMIM and GeneCard databases. Venny was used to create a Venn diagram, and 68 candidate biological targets shared by CHA and glioma were ultimately identified (**Figure 7A**). These 68 candidate targets were input into the STRING database (confidence > 0.9) and Cytoscape was used to draw a PPI network graph containing 45 targets and 77 edges (**Supplementary Figure S6**). The core targets were calculated employing 11 algorithms (see Method section) included in Cytoscape, and the effect values of each algorithm’s results were obtained. By integrating the results of various algorithms, we selected nodes that appeared in at least 10 algorithms and ranked in the top 20 in the effect values of each algorithm as core genes (**Supplementary Table S2**). Ultimately, 16 core genes were identified (**Figure 7B**), including STAT1, SRC, CASP3, NFKB1, MMP9, TLR4, MAPK8, PRKCD, PIK3R1, MAPK1, MMP2, PRKCA, IKBKB, IGF1R, ITGB2, and PDGFRB. Among them, the STAT1 gene ranked the highest in the algorithm prediction, and these results were consistent with our GSEA enrichment analysis conclusions.

The molecular docking of CHA with STAT1 was conducted using Discovery Studio to simulate their binding spatial conformation (**Figures 7C–E**). All ten docking results exhibited binding energies less than -7 kJ/mol, indicating that CHA could adapt well to the active pocket of STAT1 and bind with multiple amino acid residues. The one with the lowest binding energy -41.741 kJ/mol was selected for visualization (**Figure 7F**). The angle energy was -125.229 kJ/mol, while the bond energy was -14.3848 kJ/mol. CHA forms hydrogen bonds, which contributes most to the docking, with key residues of Lys 584, Arg 602, ALA 630, and Glu 632. These findings, from the molecular structural level, demonstrated the binding mode between STAT1 and CHA, further confirming the potential of CHA in modulating the downstream signaling of STAT1.

### 3.8 Clinical application of CHA in the treatment of recurrent glioma patients

**Figure 8:**
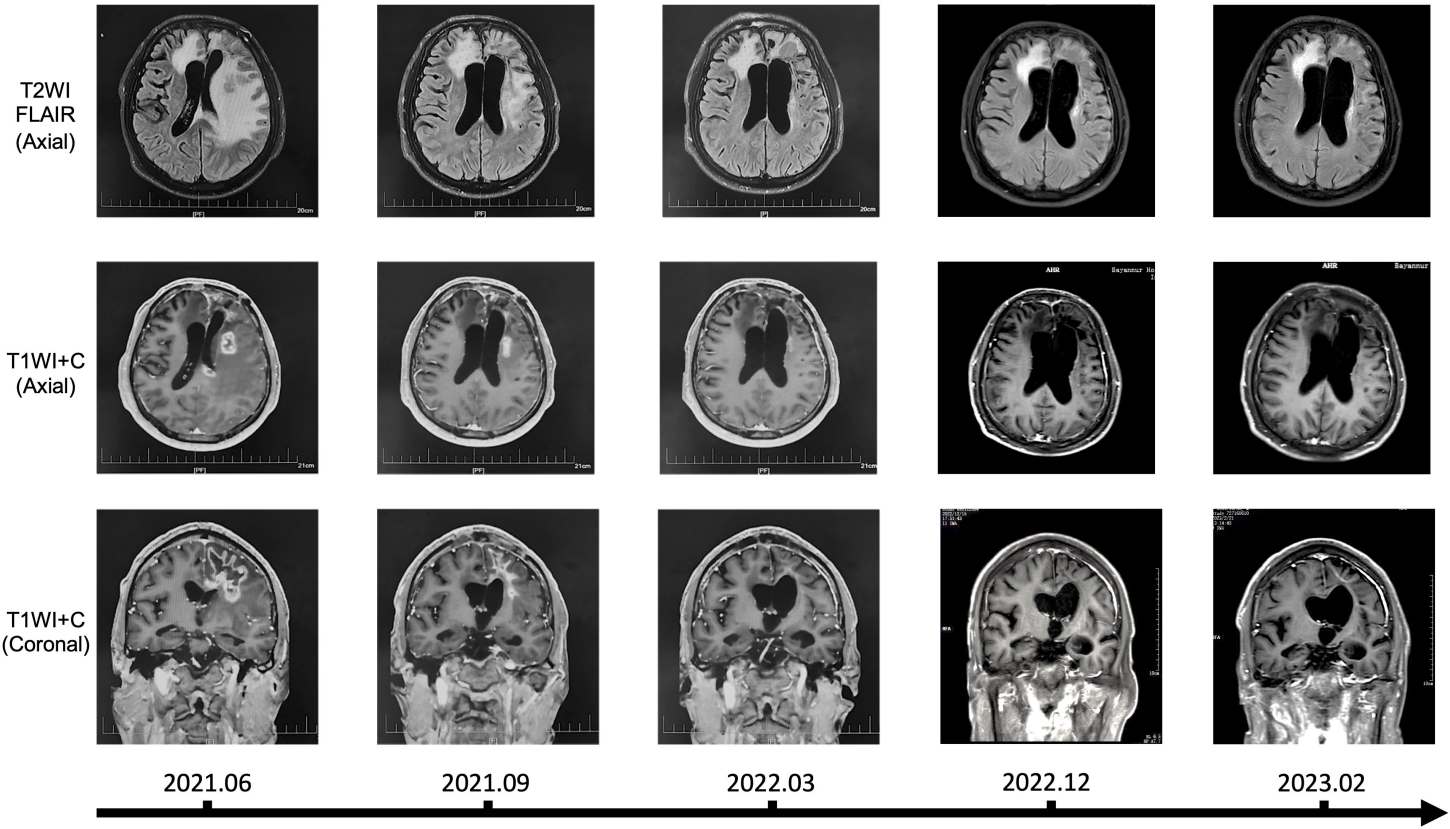
MRI findings over time in a patient treated with CHA.

We hereby reported a clinical application of CHA in the treatment of patients with recurrent glioma, along with imaging data for the first time. The patient was histologically diagnosed with WHO Grade 4 astrocytoma, IDH-mutant (IDH1 R132H mutation, MGMT promoter methylation, 1p19q codeletion, TERT C250T mutation, TERT C228T wildtype, IDH2 wildtype, and BRAF wildtype), and experienced two recurrences after surgery and concurrent chemoradiotherapy, with intolerance to TMZ during the treatment process. In June 2021, after passing a drug allergy skin test, the patient received a treatment regimen with CHA. The treatment was administered through intramuscular injection, with a dosage of 3.0mg/kg once daily. Each vial (30 mg) was dissolved in 0.5 mL of saline or sterile injectable water (with an allowable deviation of up to 10% in the actual amount of saline or injectable water used) and then injected all at once. The patient received continuous administration of CHA for 28 days, followed by a 7-day drug holiday, completing a total of 27 full cycles without interruption. Over the period from June 2021 to March 2022, the patient demonstrated good tolerance to CHA, and no hepatic or renal impairment or other serious adverse reactions were observed. Karnofsky Performance Status (KPS) score remained ≥ 70. The patient exhibited some concomitant symptoms, including dysarthria, memory and calculation decline, indistinct speech, and muscle strength of grade III in the right limbs, all of which were considered related to postoperative cerebral edema and not related to drug treatment. Long-term follow-up of the patient was conducted, and magnetic resonance imaging (MRI) was assessed according to the Response Assessment in Neuro-Oncology (RANO) criteria (**Figure 8**). Before treatment, the patient’s MRI showed a large area of signal intensity in the left frontal lobe and splenium of the corpus callosum on fluid-attenuated inversion recovery (FLAIR), with unclear boundaries and multiple irregular ring enhancements on T1 Post-Gadolinium (T1WI+C). During mid-treatment (September 2021; March 2022), the FLAIR sequences revealed signal intensity areas around the postoperative cavity, which was effectively reduced compared to pre-treatment, and the irregular enhancement range on T1WI+C also decreased markedly, with the treatment effect assessed as partial response (PR). A follow-up MRI in 2023 demonstrated further reduction of the abnormal area, with the disappearance of irregular enhancement areas on T1WI+C. The patient continued to receive CHA treatment until January 2024, with monthly follow-ups indicating good patient condition and no new symptoms during the treatment period. Afterward, the patient discontinued the medication voluntarily. In summary, our clinical data demonstrated that CHA had a good therapeutic effect against glioma, providing support for the further development of drugs for glioma treatment.

## 4 Discussion

TAMs, encompassing myeloid cells and microglia, are extensively enriched in gliomas. Myeloid cells exhibit potent suppressive properties, while microglia tend to have pro-inflammatory inclinations (37). Pseudotime analysis was used to dissect the transition from BMDMs to macrophages, a process accompanied by a decrease in the expression of IFN pathway genes and a rapid increase in the expression of the pro-angiogenic factor Vegfa. This culminates in the formation of a suppressive population characterized by high expression of Gpnmb and Apoe genes, marking the completion of the tumor’s “hijacking” of monocytes. According to traditional views, macrophages can polarize into M1 and M2 types, but the new perspectives widely regard macrophage polarization as a continuous process (8). As anticipated, the TAM3 subset (Gpnmb+ and Apoe+) is more similar to the previously recognized M2 macrophages, while the TAM1 subset resembles the M1 type but still expresses a subset of tumor-suppressive genes, reflecting the broad heterogeneity of this population. Microglia surrounding the tumor exhibit activated IFN pathway and enhanced MHC-II expression, and display active transcription factors and cytokines during their activation process; these findings are consistent with Ochoa’s conclusions (37, 38). Therefore, microglia may be an effective force against tumors in the early stages but as the number of blood-derived NK cells, T cells, BMDMs, and other cells increases, they may gradually “decline”, and lose their ability to resist tumors. However, further experimental evidence is needed to elucidate how tumors “hijacking” the immune microenvironment.

CHA has extensive biological activities, including antimicrobial activity, anti-autoimmune disease, and metabolic regulation (40–42). Early studies have primarily focused on the direct cytotoxicity of CHA. Gupta et al. have systematically reviewed the mechanisms of CHA’s antitumor effects, including inducing apoptosis, oxidative stress, affecting tumor’s proliferation and migration, and anti-angiogenesis; however, more recent studies have begun to focus on the immunomodulatory functions and anticancer mechanisms of CHA (11). Our research, through a series of animal model experiments, revealed the key role of CHA in regulating the function of macrophages and microglia. Our early findings indicated that CHA could promote the repolarization of M2-type macrophages to the M1 phenotype, with CHA modulating macrophage polarization by promoting and inhibiting the activation of STAT1 and STAT6 pathways, respectively (16). We observed that CHA could notably extend the survival of model mice and exert its antitumor effects by regulating the state of TAMs and microglia. GSEA showed that CHA activated a series of gene pathways closely related to adaptive immune responses. Notably, CHA remarkably enhanced the antigen-presenting function of macrophages and the expression of T-cell immune activation-related genes, demonstrating a high degree of congruence with adaptive immunity. Our previous target predictions have suggested that the targets of CHA on glioma cells may be related to the JAK-STAT and NF-κB pathways (43). Li et al. have found that in the melanoma model mice treated with CHA, the proportion of M2-TAMs and CD4-Foxp3^+^ T cells is significantly decreased, while the proportion and activity of CD8^+^ T cells and M1-TAMs are markedly increased (44). This involves a mechanism closely related to the JAK-pSTAT1-IRF1 pathway (18). Additionally, CHA can reduce STAT3 phosphorylation and STAT3 and Snail protein levels, block the STAT3/Snail pathway, inhibit the progression of osteosarcoma cells, and induce apoptosis (45). All these conclusions imply that the JAK-STAT pathway is a key target of CHA in tumor treatment. Through scRNA-seq, PPI network analysis, and molecular docking, the present study proposed for the first time that CHA activated microglia through STAT1. STAT1 is a key transcription factor extensively involved in the IFN signaling pathways and JAK-STAT signaling pathways, playing a crucial role in regulating immune cell activation, proliferation, and differentiation. Therefore, we believe that improving the function of microglia could potentially benefit patients.

CHA now possesses the potential for clinical application. Our patient data demonstrated that CHA had good safety, and also showed remarkable therapeutic efficacy. There are currently multiple ongoing clinical trials investigating the use of CHA for cancer treatment (NCT03751592, NCT02245204, and NCT02136342). Our center has initiated a Phase I clinical trial (CTR20160113) on the treatment of advanced glioma with CHA, and its reliable safety and certain therapeutic effects have been proven. CHA is currently the subject of a “Phase II/III study evaluating the safety and efficacy of injectable CHA in the treatment of recurrent grade IV glioblastoma” (CTR20181644), which may provide more reliable evidence for the clinical efficacy of CHA.

## 5 Conclusions

In summary, we can conclude that the JAK-STAT pathway is the core molecular link between myeloid cells, microglia in the glioma microenvironment, and the action mechanism of CHA. Our findings refine the details of how CHA modulates immunity to treat glioma at the RNA and single-cell levels and demonstrate promising antitumor efficacy in animal models. Our case data also highlight the immense potential of CHA for clinical application. This research outcome provides robust support and new insights for drug development and further lays a theoretical foundation for the clinical development of CHA treatment for glioma patients.

## Data Availability

The data presented in this study are deposited in the OMIX repository, accession number OMIX008544.
