# Supplementary figures and images for: ScRNA-seq unveils the functional characteristics of glioma-associated macrophages and the regulatory effects of chlorogenic acid on the immune microenvironment—a study based on mouse models and clinical practice
Source: Front Immunol. 2025 Jan 10;15:1494806. doi: 10.3389/fimmu.2024.1494806 (PMC11757274; doi:10.3389/fimmu.2024.1494806)

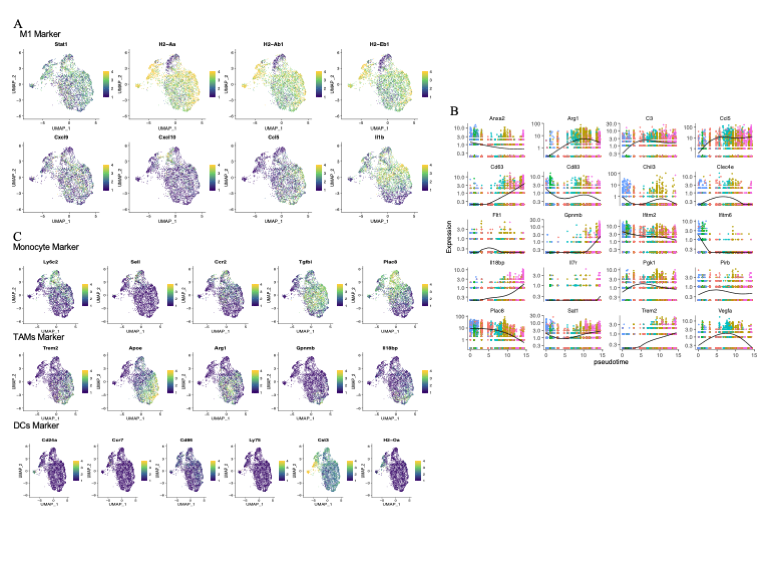

Supplement: Supplementary file 1 [file DataSheet1.zip › Supplementary Material /Figure S3.tiff]

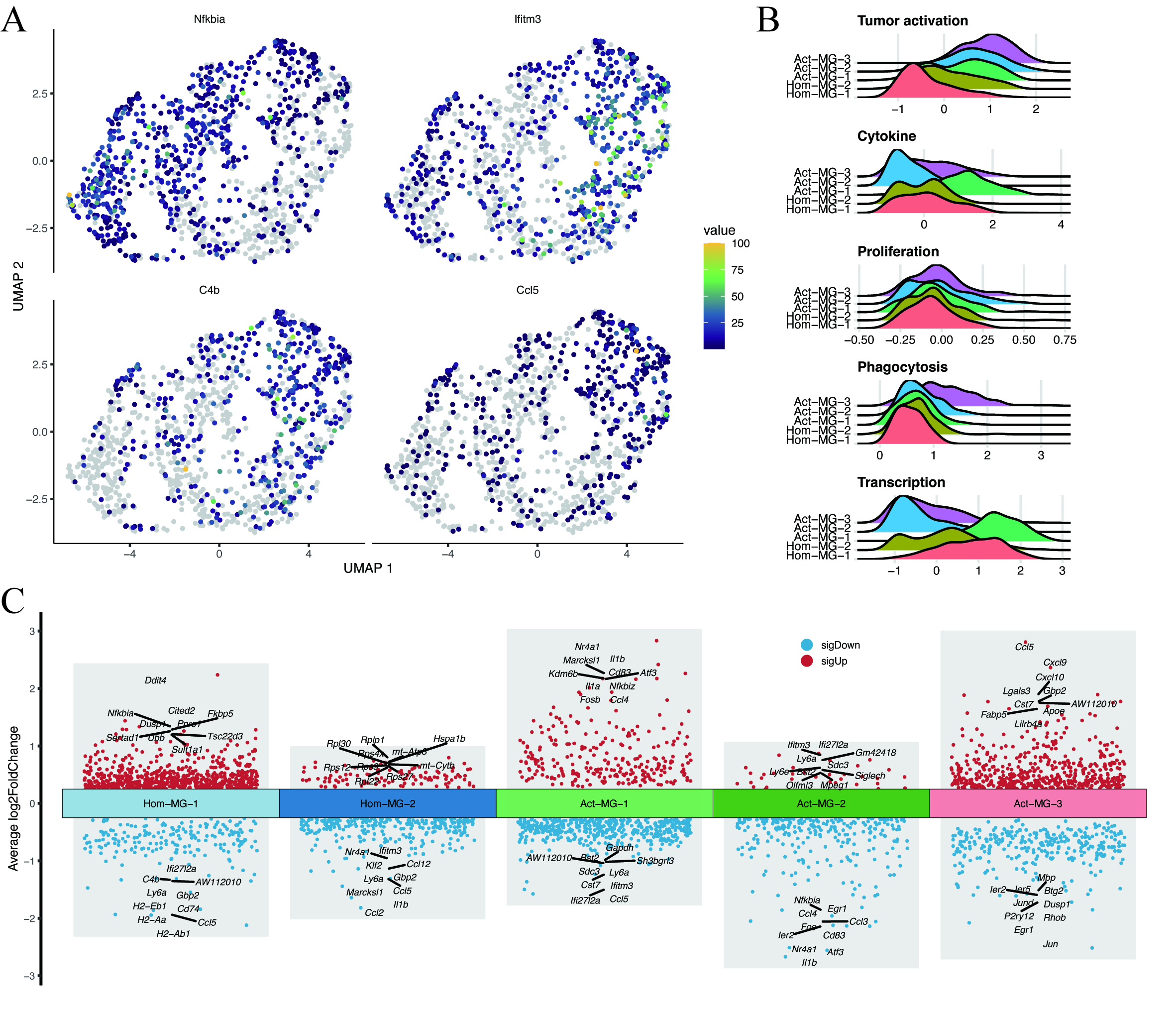

Supplement: Supplementary file 1 [file DataSheet1.zip › Supplementary Material /Figure S4.tif]

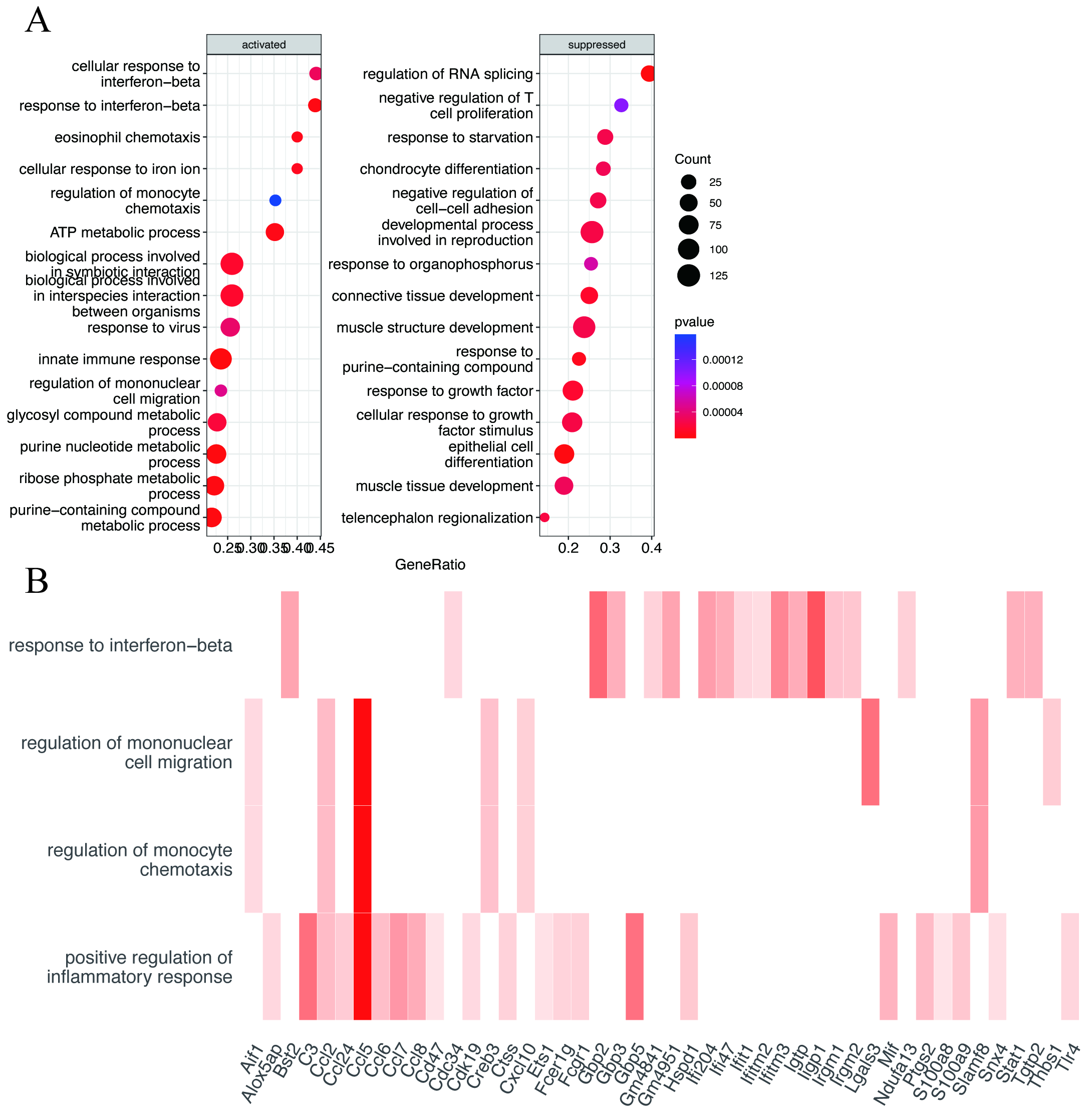

Supplement: Supplementary file 1 [file DataSheet1.zip › Supplementary Material /Figure S5.tif]

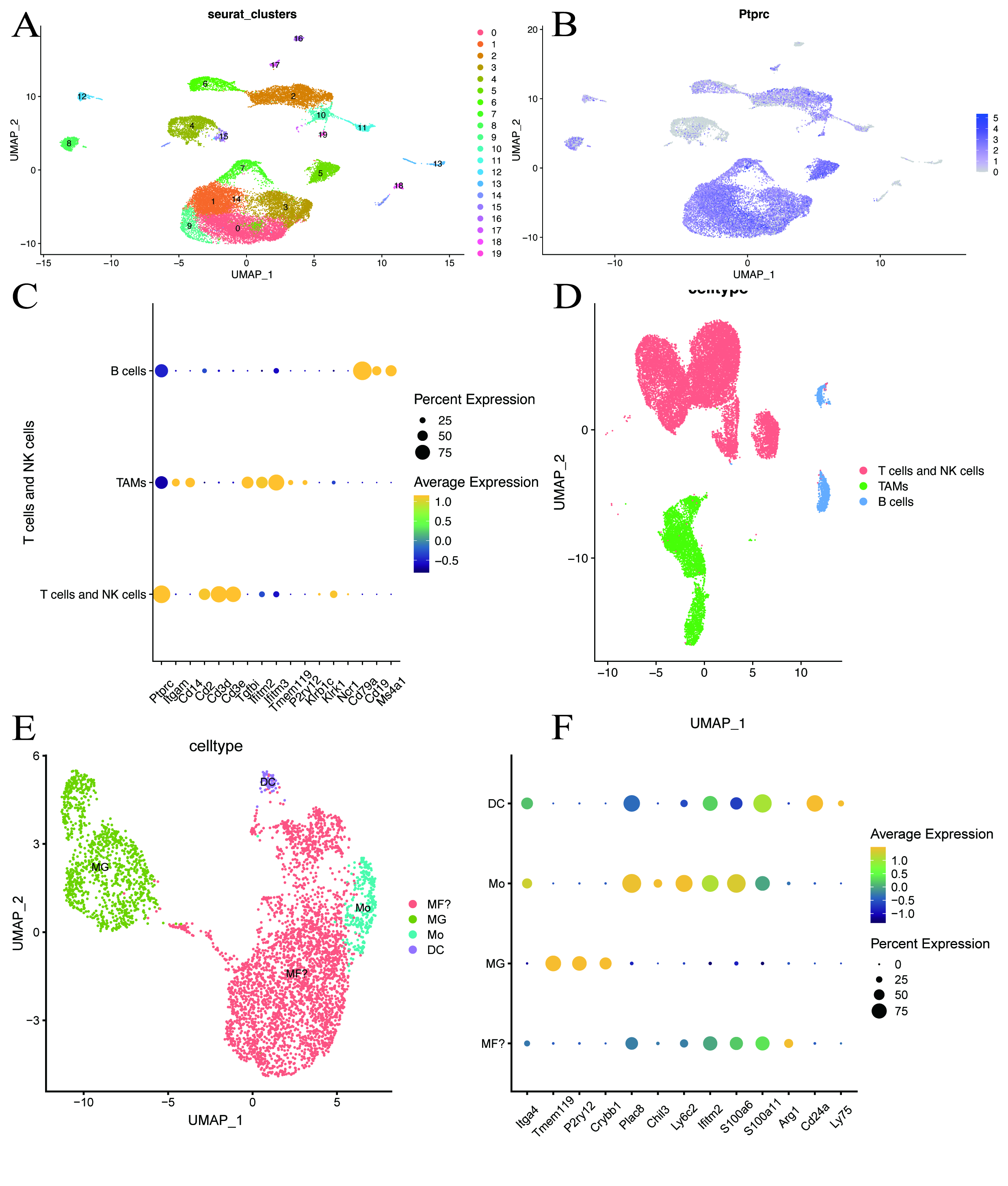

Supplement: Supplementary file 1 [file DataSheet1.zip › Supplementary Material /Figure S2.tif]

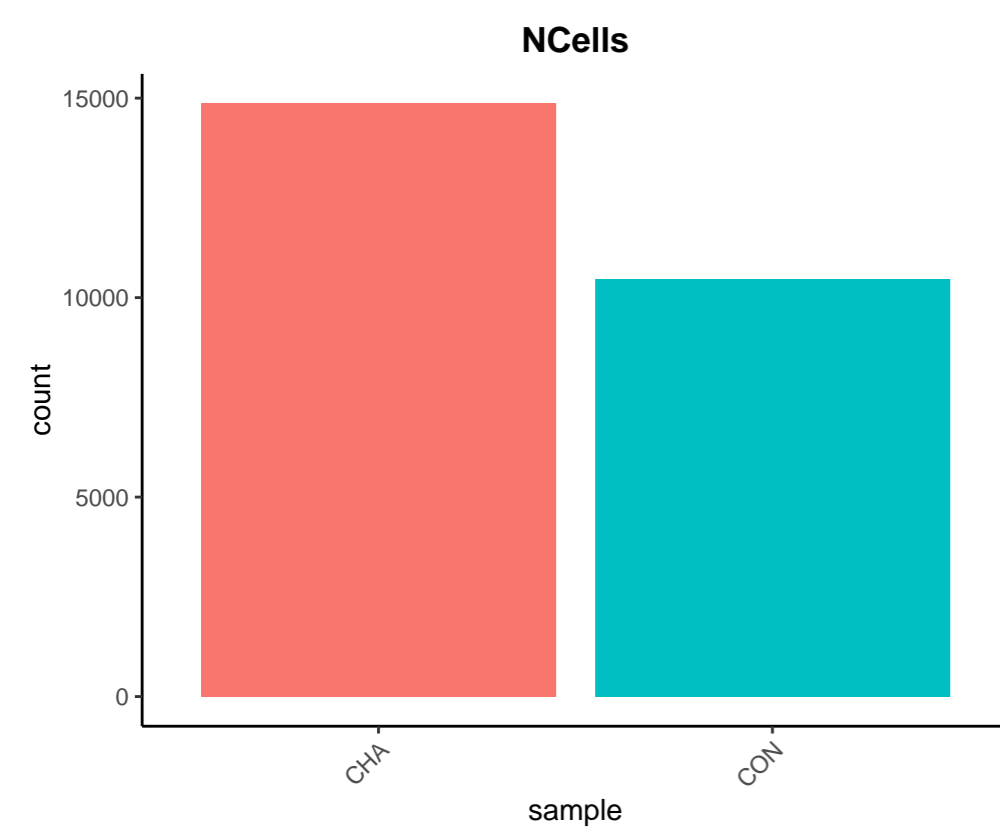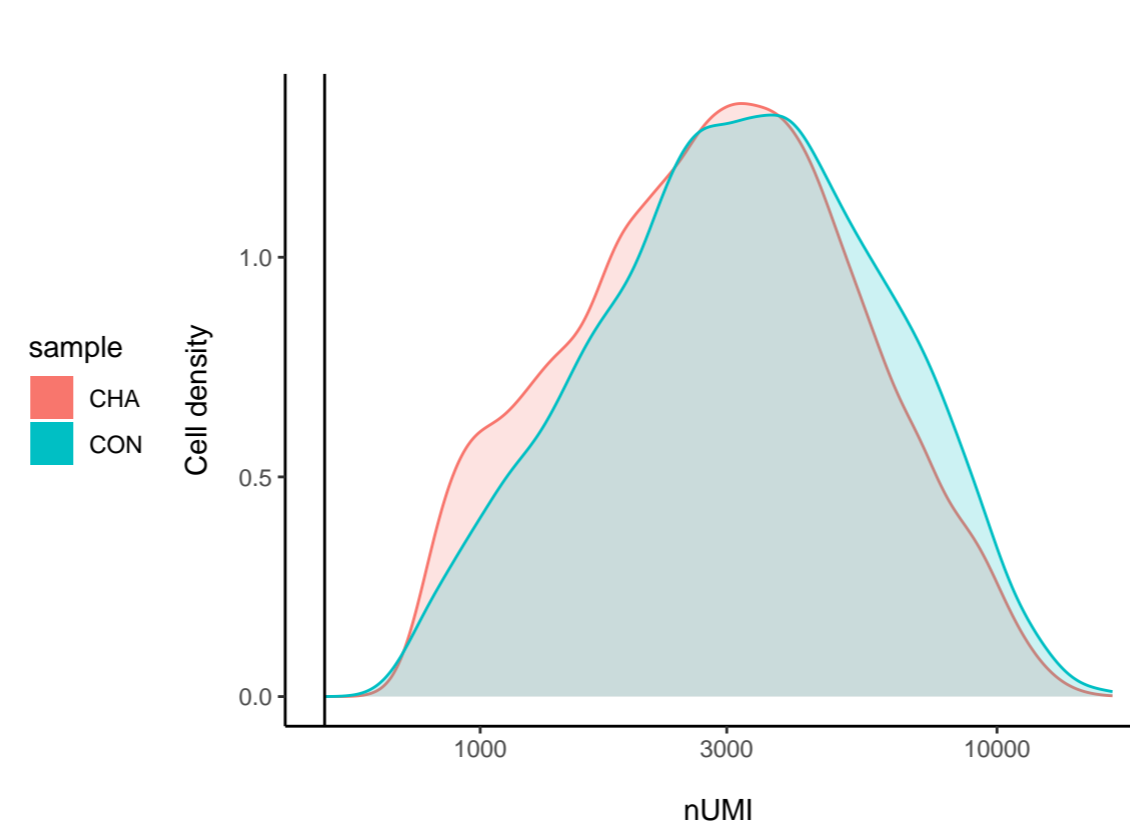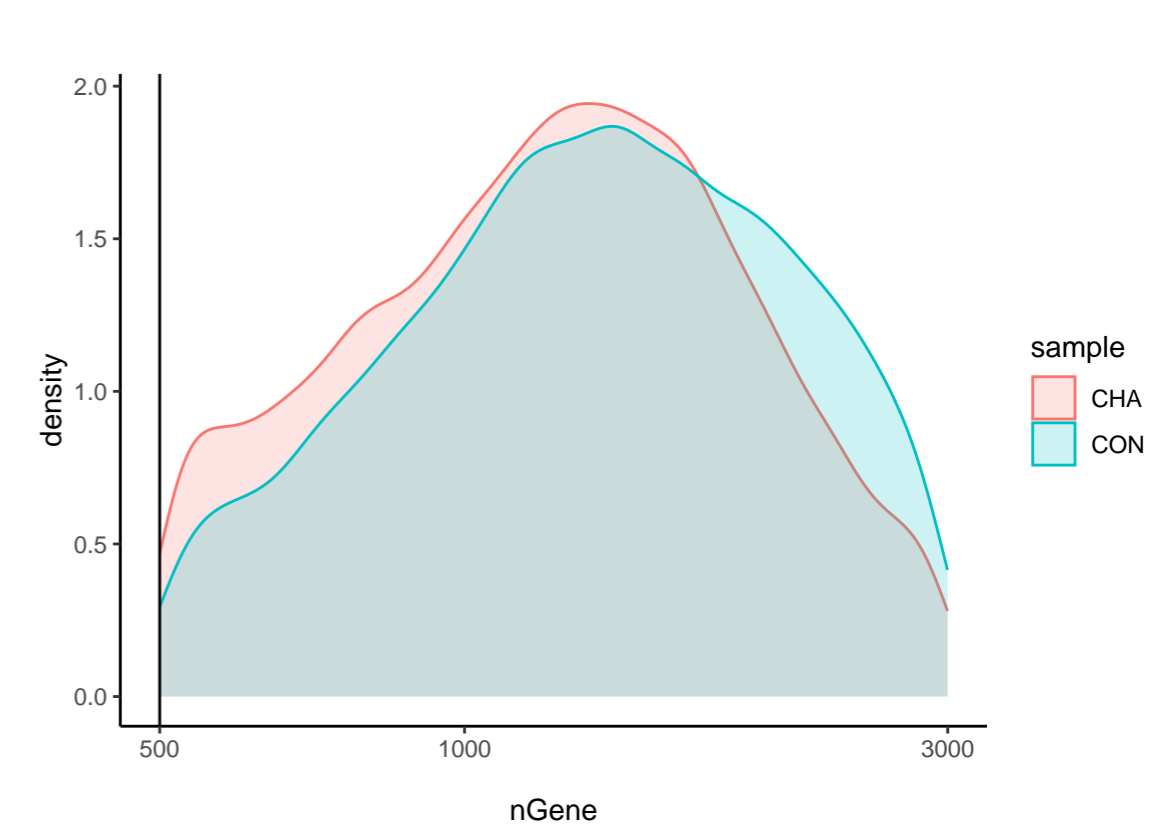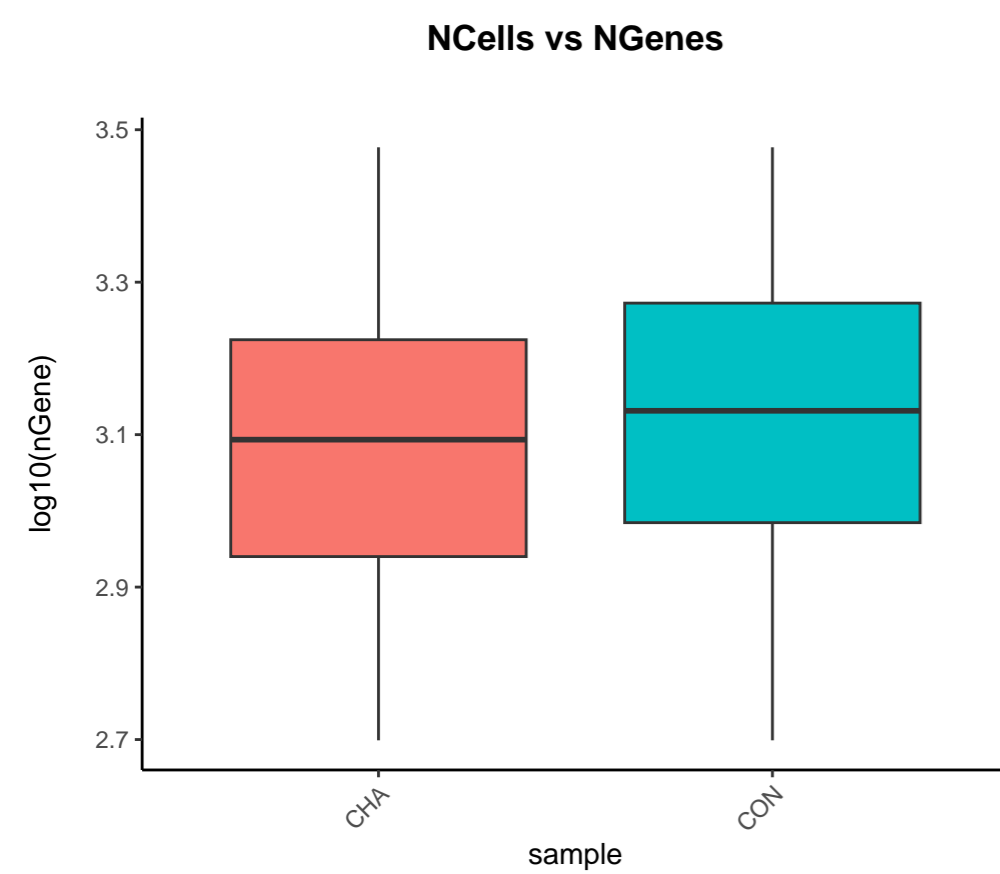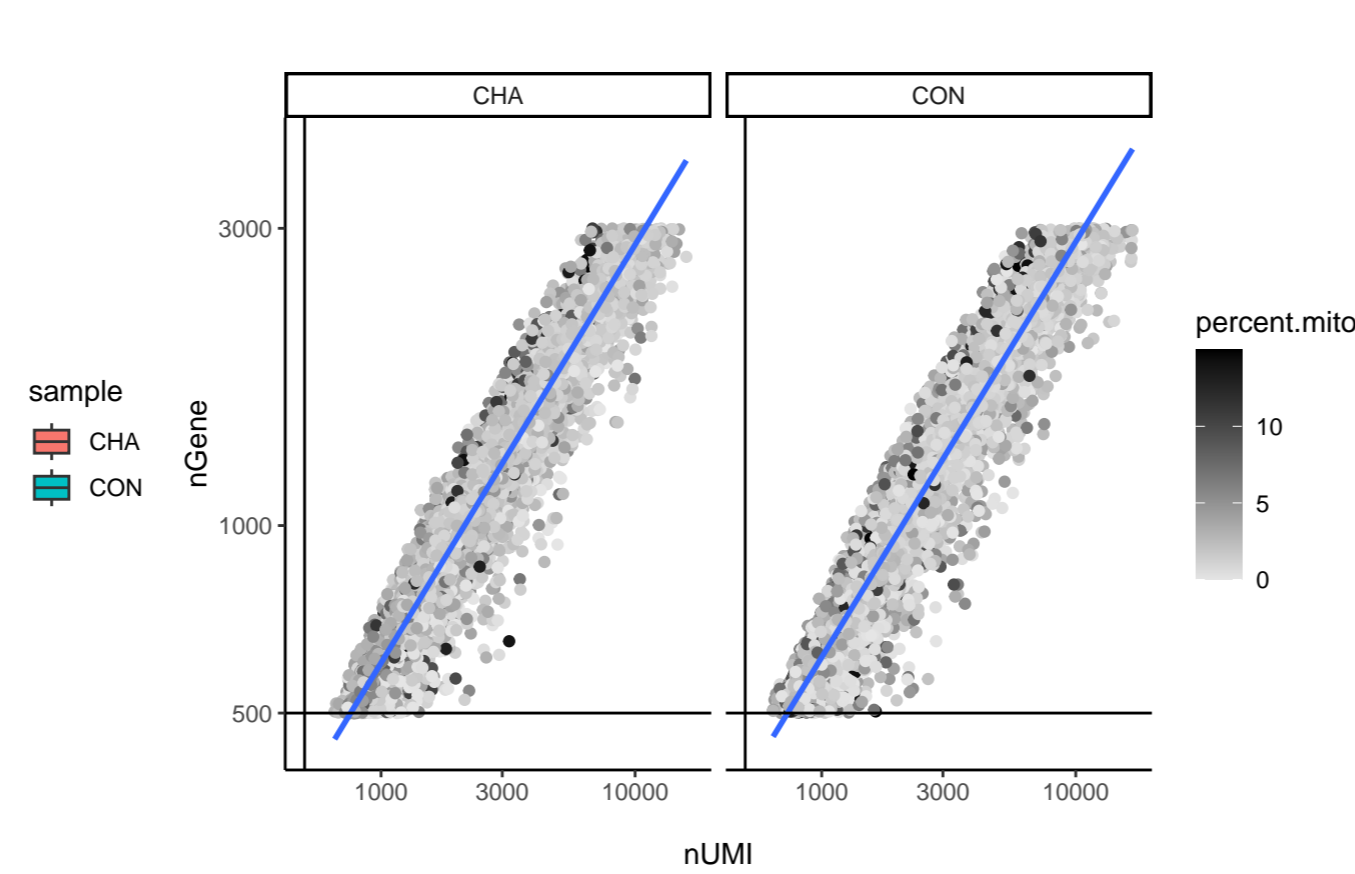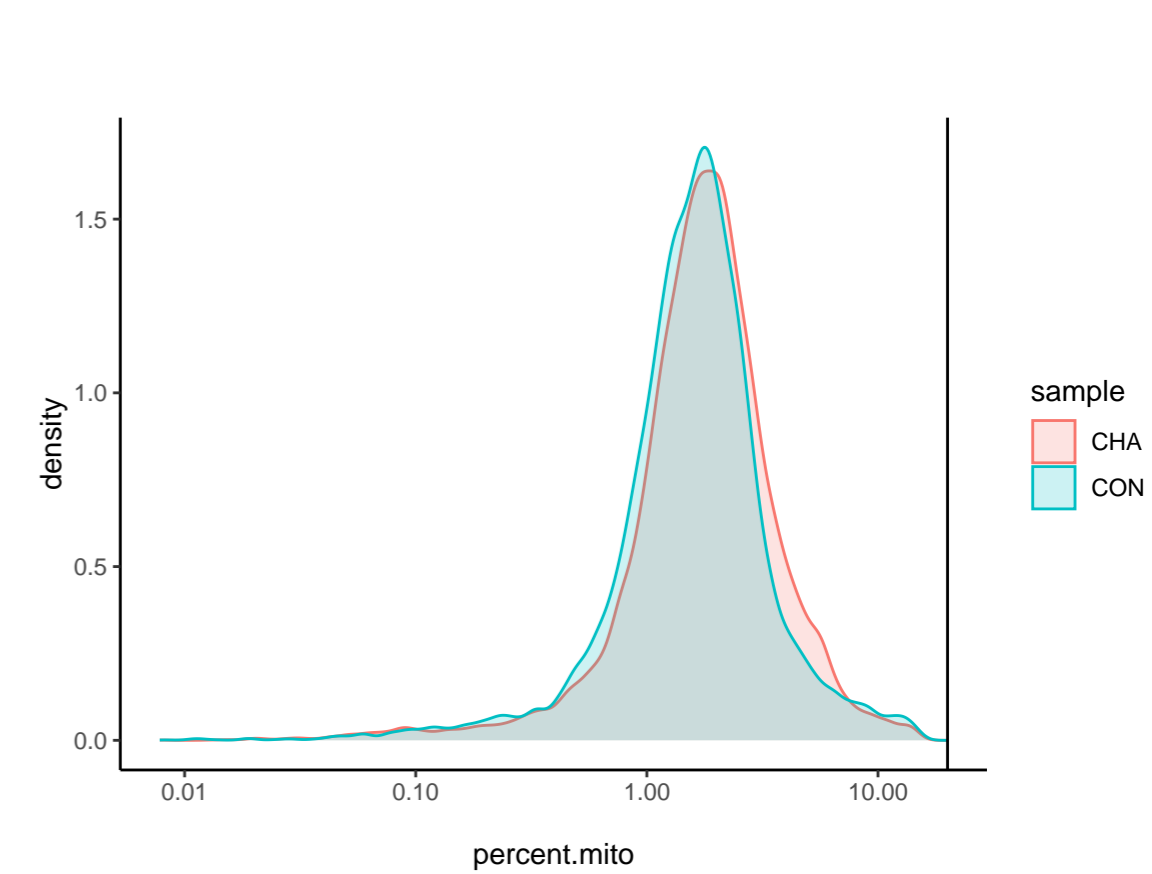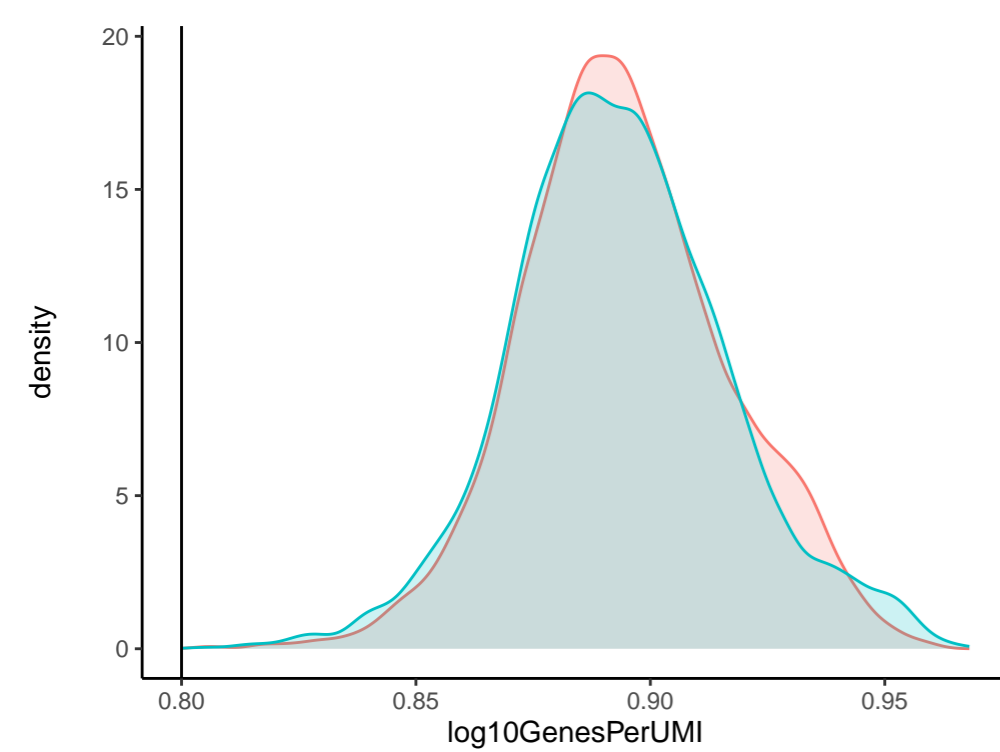

Supplement: Supplementary file 1 [file DataSheet1.zip › Supplementary Material /Figure S1.pdf]

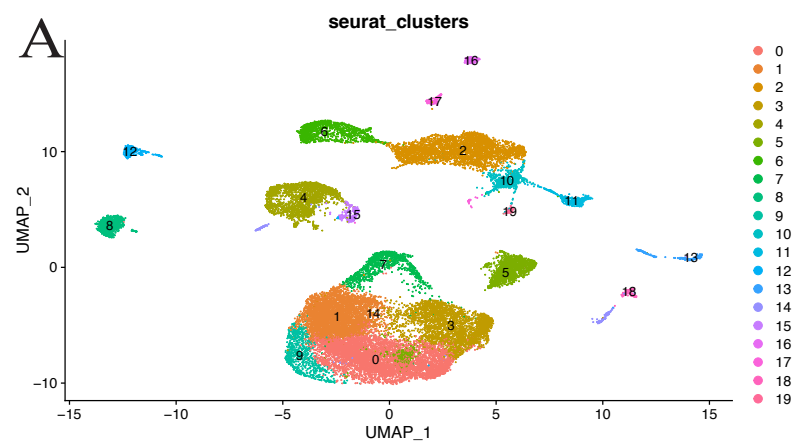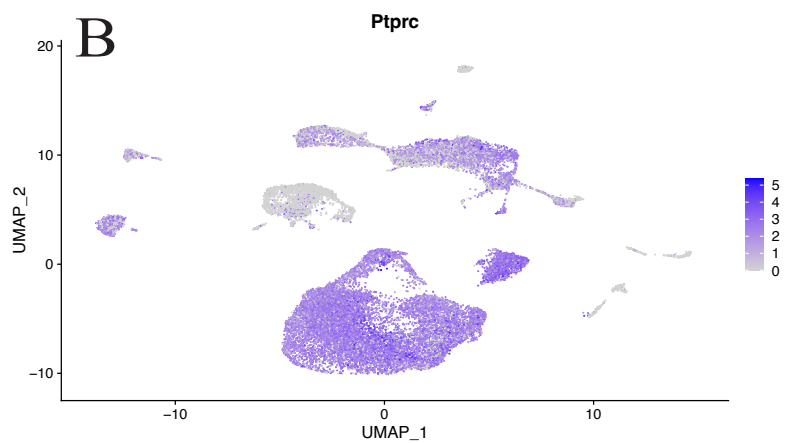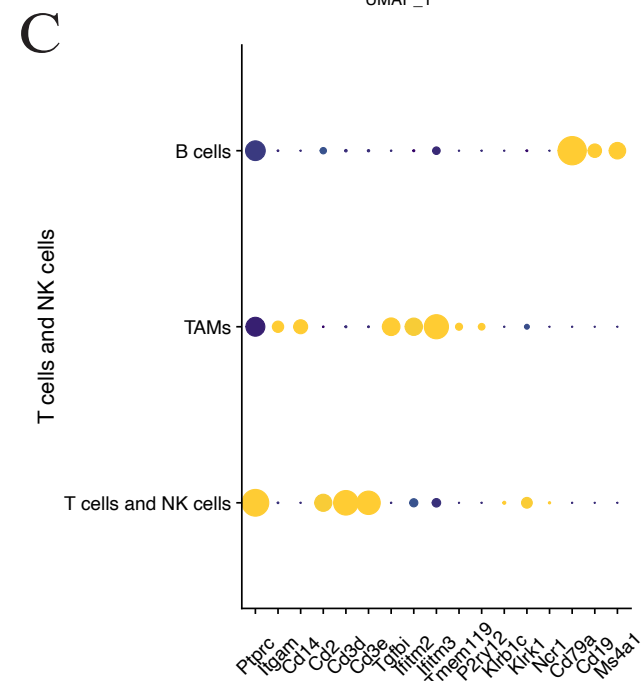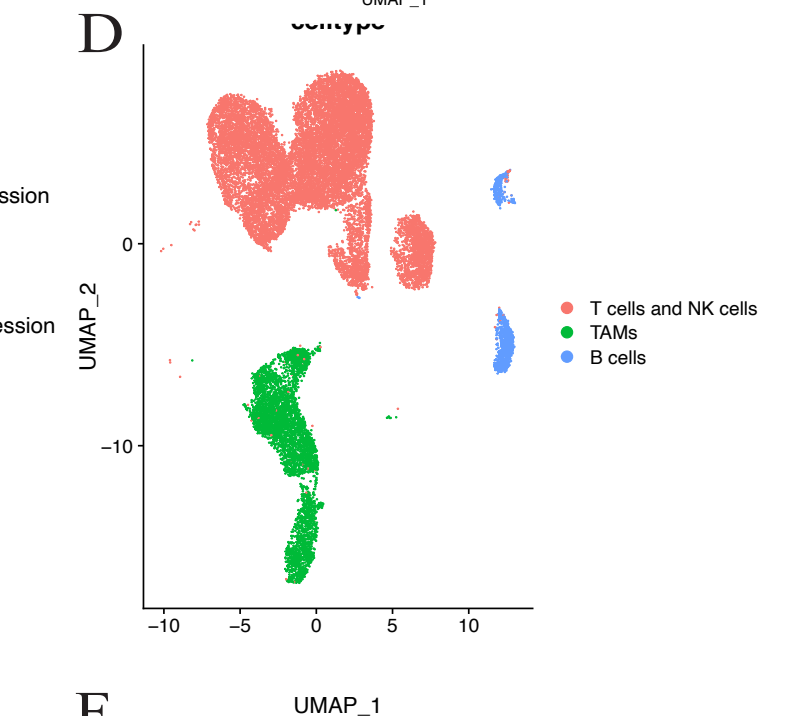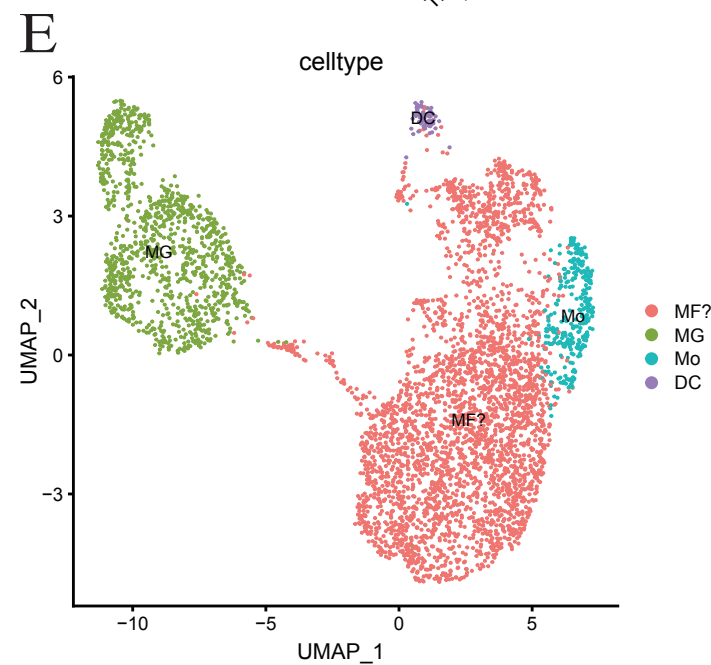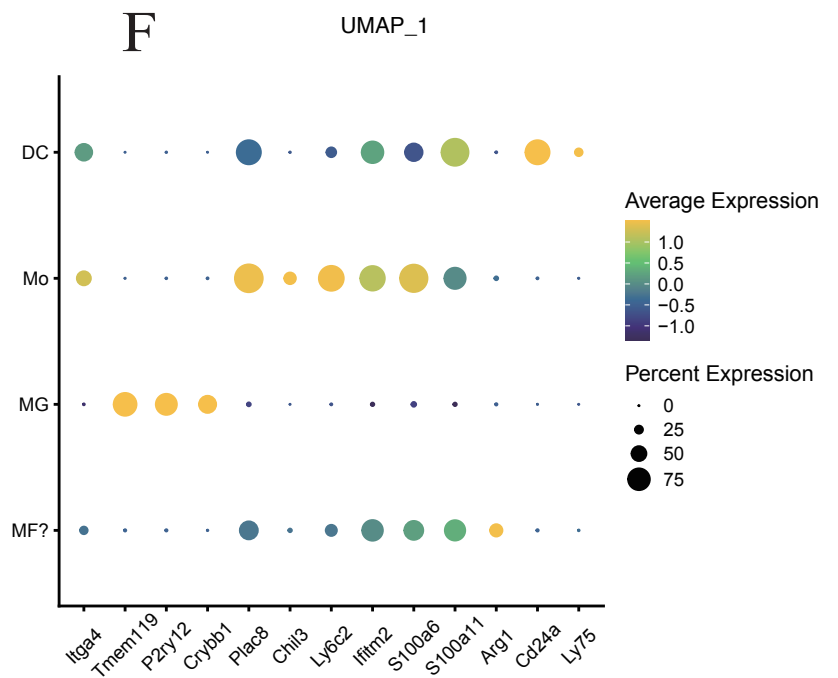

Supplement: Supplementary file 1 [file DataSheet1.zip › Supplementary Material /Figure S2.pdf]

A

## M1 Marker

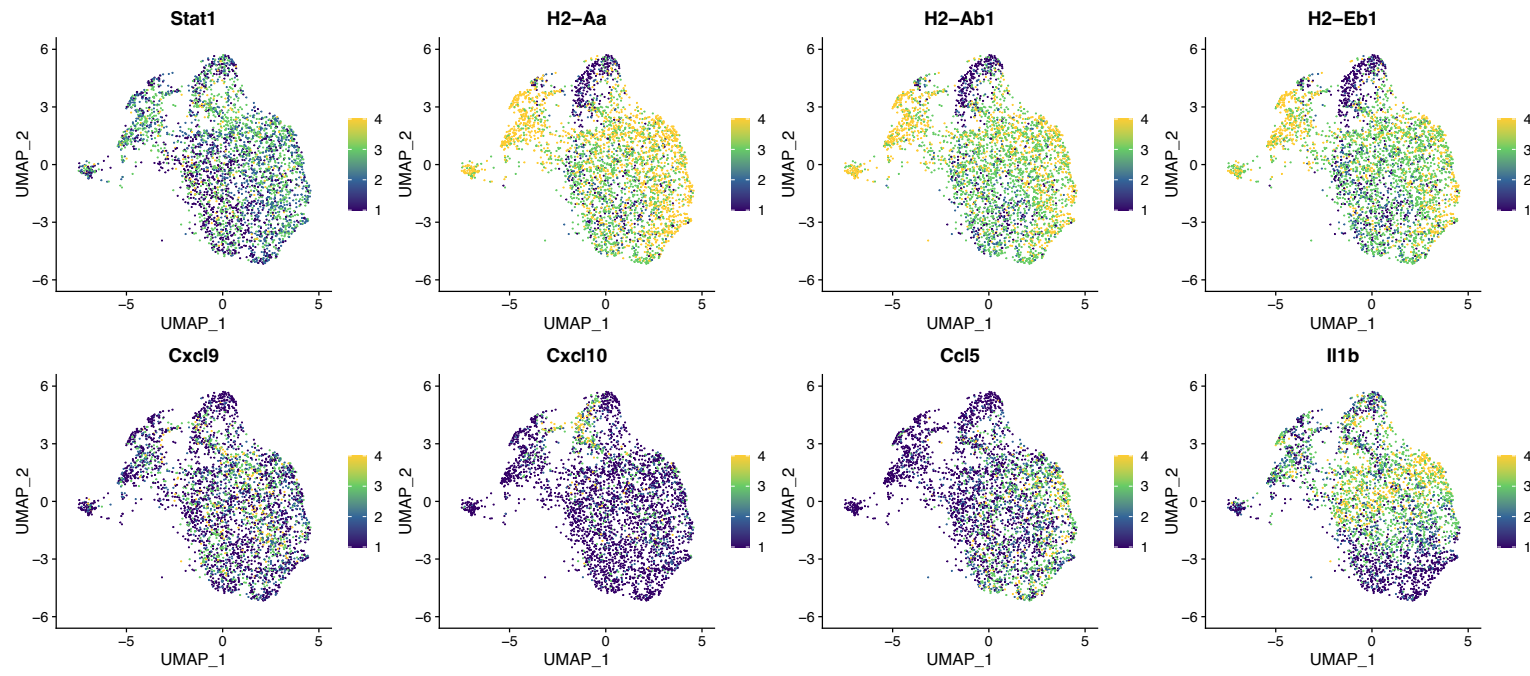

C

## Monocyte Marker

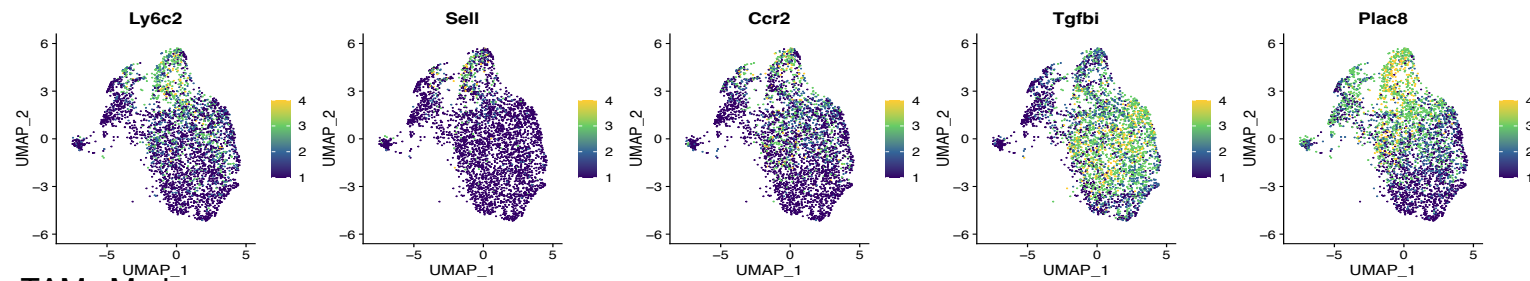

## TAMs Marker

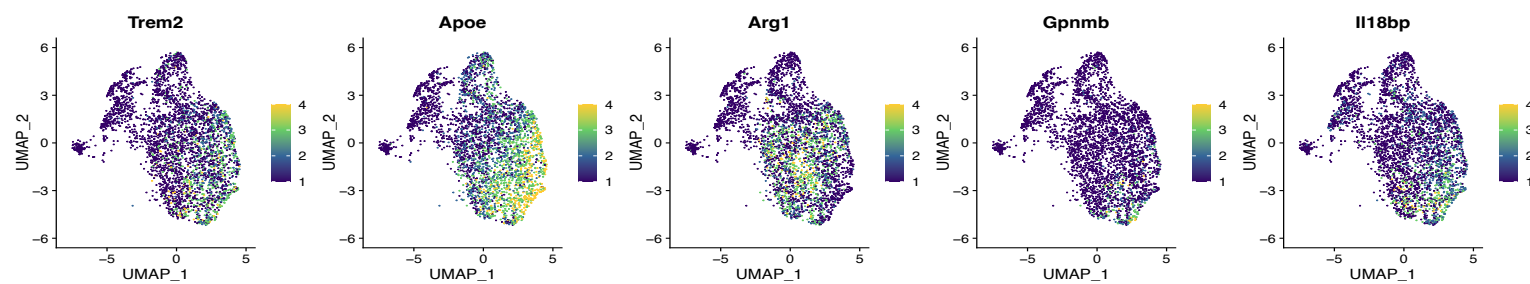

## DCs Marker

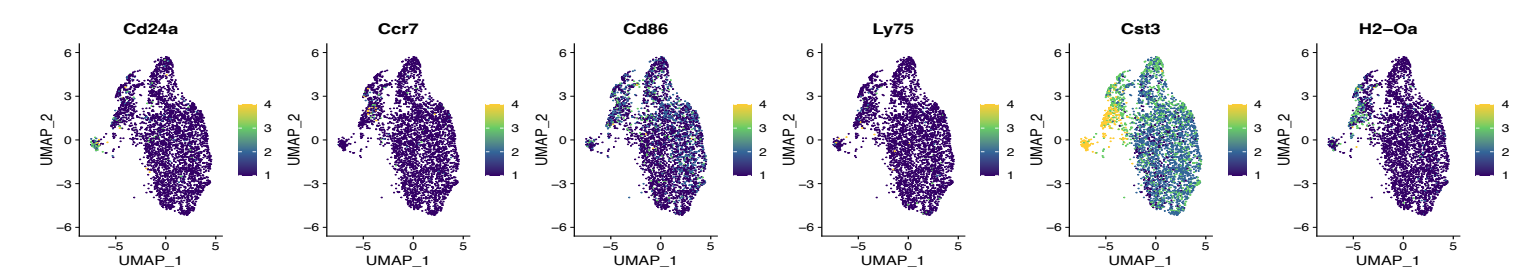

B

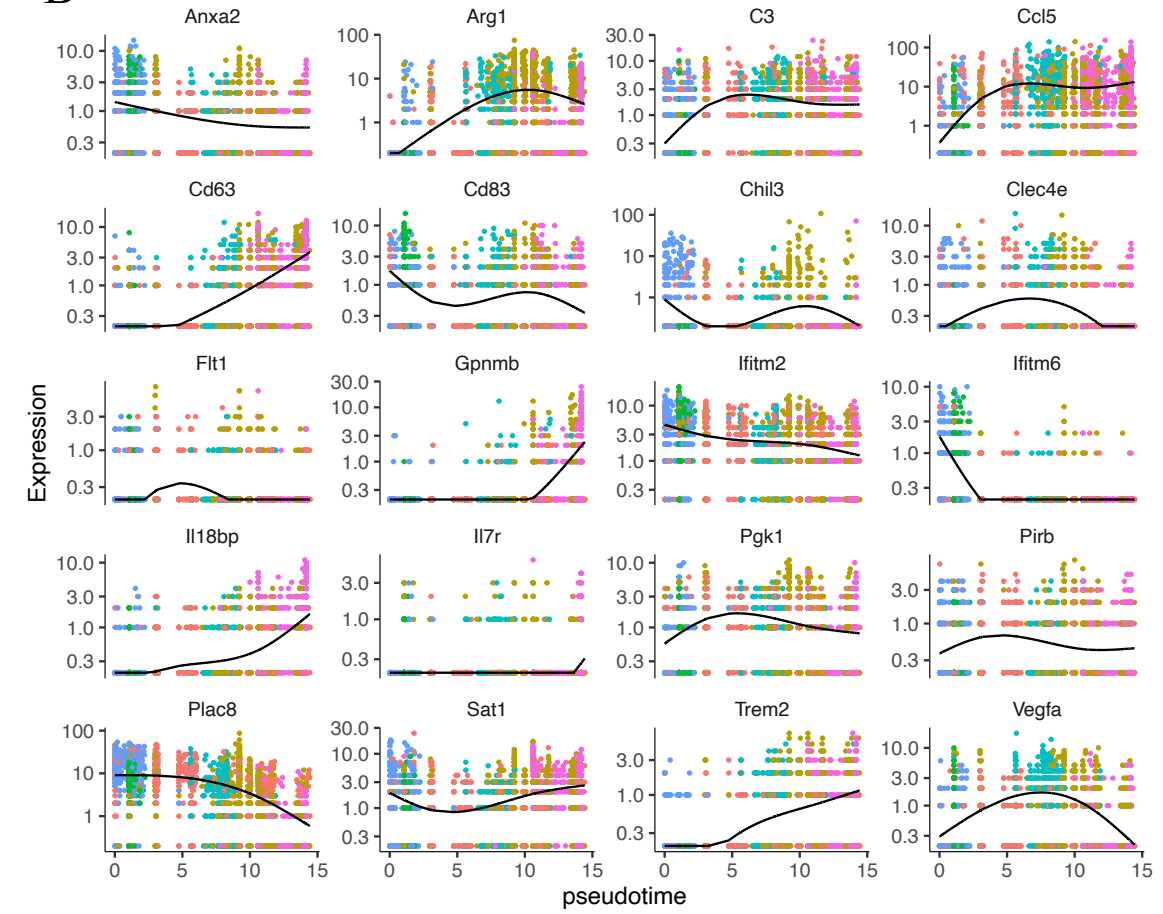

Supplement: Supplementary file 1 [file DataSheet1.zip › Supplementary Material /Figure S3.pdf]

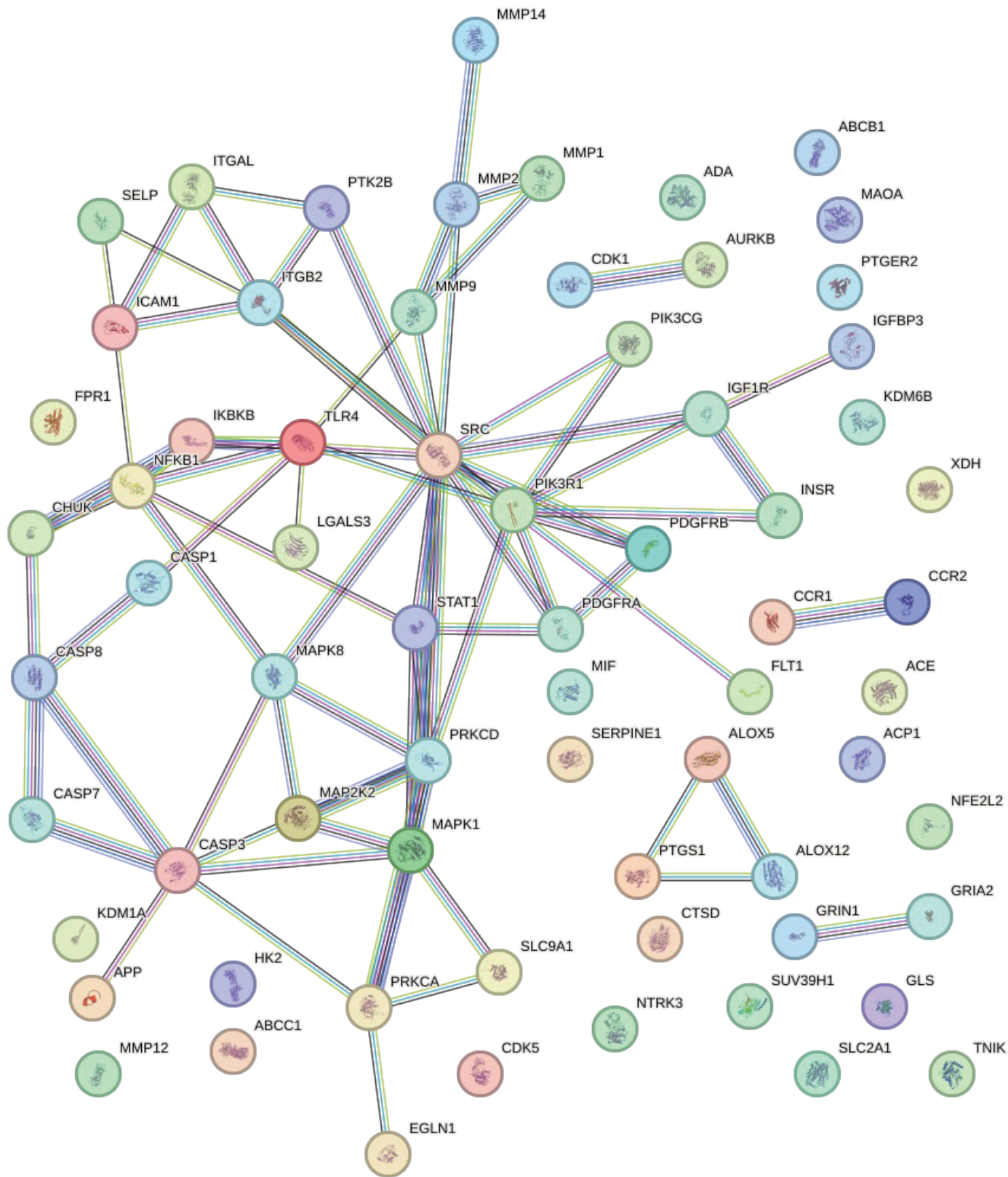

Supplement: Supplementary file 1 [file DataSheet1.zip › Supplementary Material /Figure S6.pdf]

A

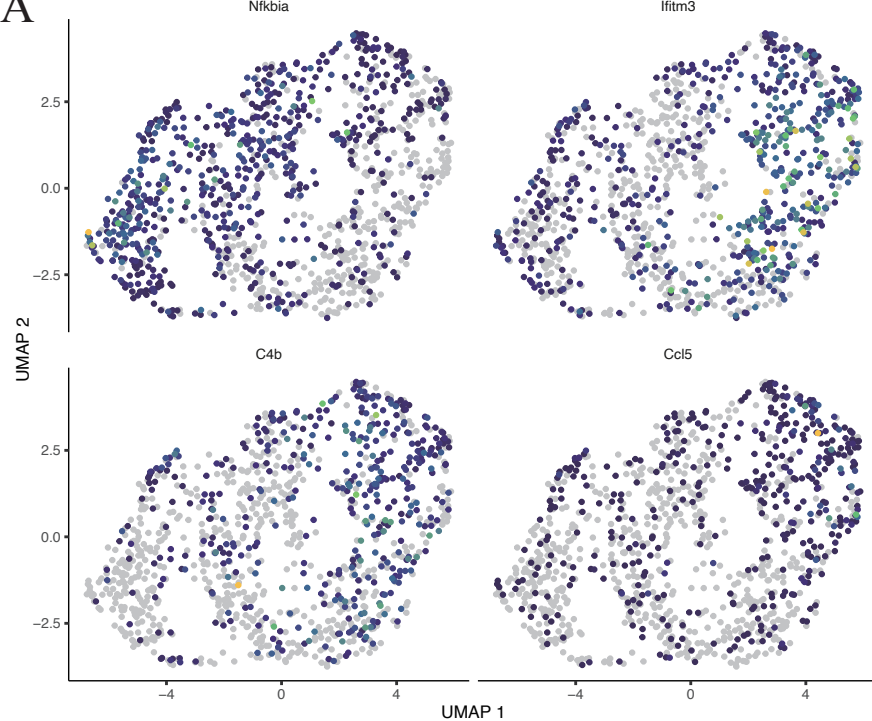

B

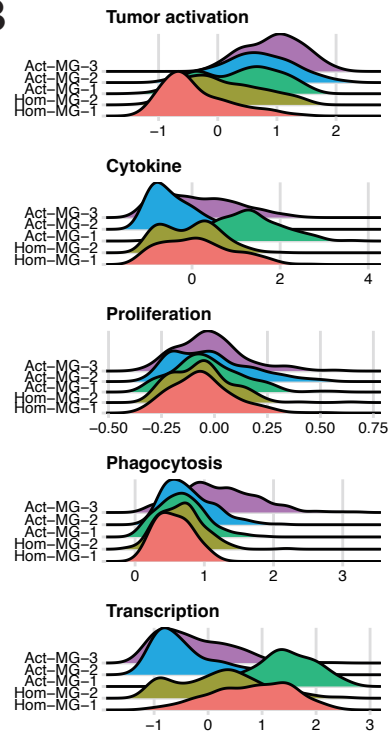

C

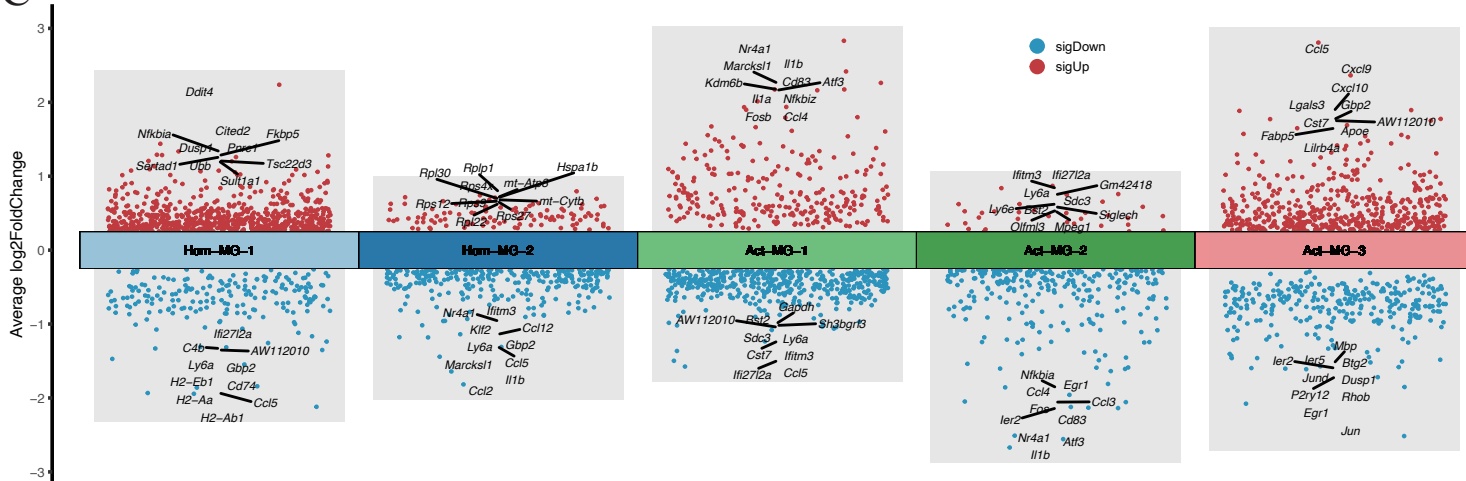

Supplement: Supplementary file 1 [file DataSheet1.zip › Supplementary Material /Figure S4.pdf]

A

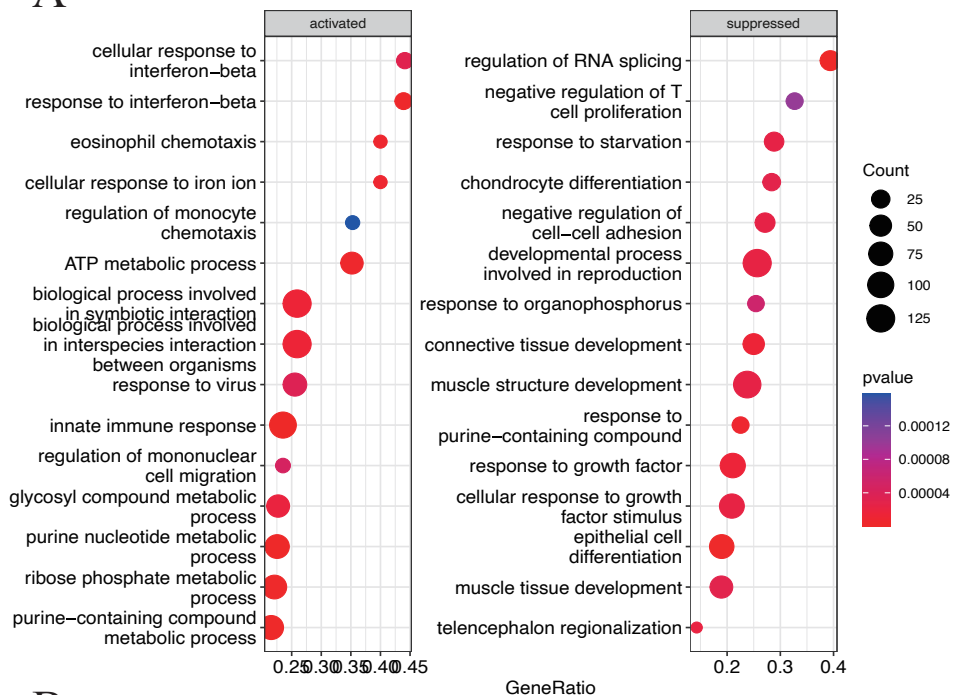

B

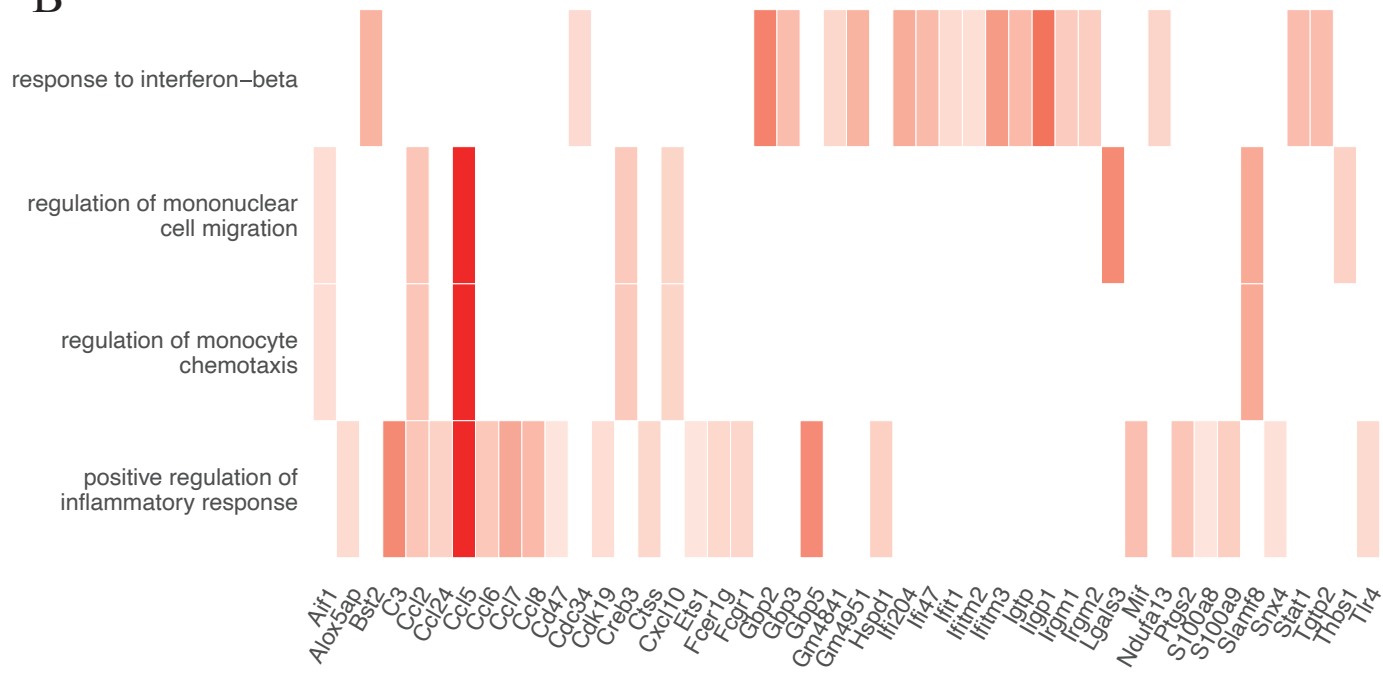

Supplement: Supplementary file 1 [file DataSheet1.zip › Supplementary Material /Figure S5.pdf]
